# Supplementary material for: RNA Modification-Related Genetic Variants in Genomic Loci Associated with Bone Mineral Density and Fracture
Source: Genes (Basel). 2022 Oct 18;13(10):1892. doi: 10.3390/genes13101892 (PMC9601451; doi:10.3390/genes13101892)
Supplement: Supplementary file 1 [file genes-13-01892-s001.zip › Figure S1.pptx]

## Slide 1
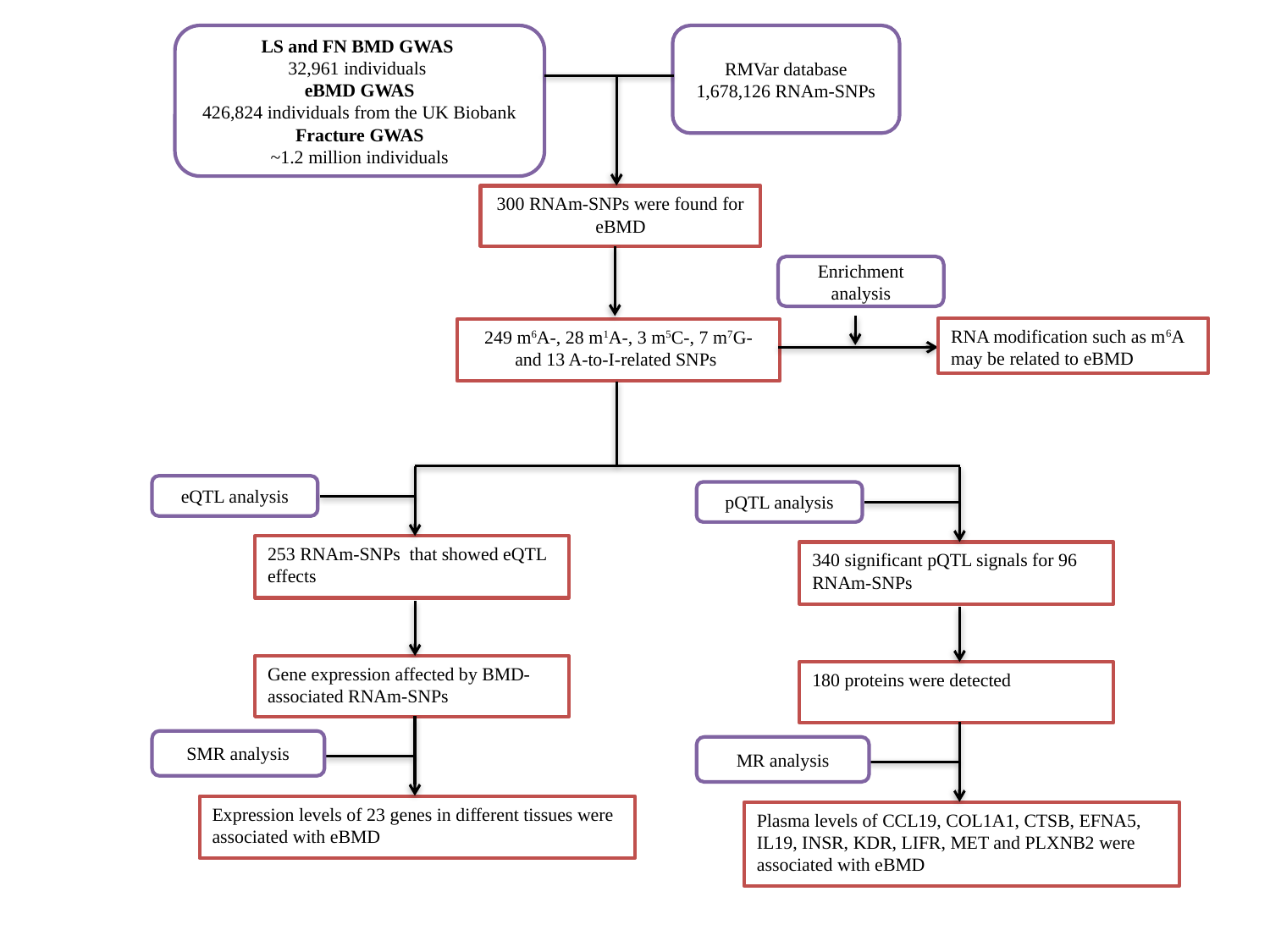

LS and FN BMD GWAS
32,961 individuals
eBMD GWAS
426,824 individuals from the UK Biobank
Fracture GWAS
~1.2 million individuals
RMVar database
1,678,126 RNAm-SNPs
300 RNAm-SNPs were found for eBMD
249 m6A-, 28 m1A-, 3 m5C-, 7 m7G- and 13 A-to-I-related SNPs
Enrichment analysis
RNA modification such as m6A may be related to eBMD
eQTL analysis
253 RNAm-SNPs that showed eQTL effects
Gene expression affected by BMD-associated RNAm-SNPs
SMR analysis
Expression levels of 23 genes in different tissues were associated with eBMD
pQTL analysis
340 significant pQTL signals for 96 RNAm-SNPs
180 proteins were detected
MR analysis
Plasma levels of CCL19, COL1A1, CTSB, EFNA5, IL19, INSR, KDR, LIFR, MET and PLXNB2 were associated with eBMD
