# Supplementary material for: RNA Modification-Related Genetic Variants in Genomic Loci Associated with Bone Mineral Density and Fracture
Source: Genes (Basel). 2022 Oct 18;13(10):1892. doi: 10.3390/genes13101892 (PMC9601451; doi:10.3390/genes13101892)
Supplement: Supplementary file 1 [file genes-13-01892-s001.zip › Supplementary_tables.pdf]

Supplementary Table S1 RNAm_SNPs identified for eBMD and fracture

SNP	rm_id	Modification type	CHR	Position	Gene	Gene region	Confidence level	Modification function	Allele1	Allele2	Allele1 freq	beta	se	P value	Trait
rs7513688	RMVar_ID_1594463	m7G	1	6592685	KLHL21	3_UTR	MeRIP-seq:(Medium)	Functional Loss	G	A	0.6399	-0.0152	0.0019	1.30E-12	eBMD
rs7513688	RMVar_ID_1559152	m7G	1	6592685	KLHL21	3_UTR	MeRIP-seq:(Medium)	Functional Loss	G	A	0.6399	-0.0152	0.0019	1.30E-12	eBMD
rs3174820	RMVar_ID_67234	m1A	1	6633037	THAP3	CDS	m1A-quant-seq:(High)	Functional Loss	A	G	0.6501	0.0183	0.0019	1.30E-20	eBMD
rs3174820	RMVar_ID_110926	m1A	1	6633037	THAP3	CDS	m1A-quant-seq:(High)	Functional Loss	A	G	0.6501	0.0183	0.0019	1.30E-20	eBMD
rs2294532	RMVar_ID_747267	m6A	1	6634866	THAP3	3_UTR	miCLIP:(High)	Functional Loss	C	T	0.6675	-0.0166	0.0019	2.30E-14	eBMD
rs2294532	RMVar_ID_270567	m6A	1	6634866	DNAJC11	3_UTR	miCLIP:(High)	Functional Loss	C	T	0.6675	-0.0166	0.0019	2.30E-14	eBMD
rs2791649	RMVar_ID_1143645	m6A	1	11072504	EXOSC10	intron	Prediction:(Low)	Functional Loss	A	G	0.2547	-0.0188	0.0021	2.20E-14	eBMD
rs11121702	RMVar_ID_1143791	m6A	1	11233087	MTOR	intron	Prediction:(Low)	Functional Gain	C	T	0.2681	-0.0180	0.0021	1.20E-12	eBMD
rs1048334	RMVar_ID_1161447	m6A	1	16014263	HSPB7	3_UTR	Prediction:(Low)	Functional Loss	T	C	0.4101	0.0106	0.0019	1.40E-08	eBMD
rs1048334	RMVar_ID_1161446	m6A	1	16014263	HSPB7	3_UTR	Prediction:(Low)	Functional Loss	T	C	0.4101	0.0106	0.0019	1.40E-08	eBMD
rs1048238	RMVar_ID_1161454	m6A	1	16015152	HSPB7	3_UTR	Prediction:(Low)	Functional Loss	C	T	0.4284	0.0113	0.0019	4.10E-09	eBMD
rs1048238	RMVar_ID_1161453	m6A	1	16015152	HSPB7	3_UTR	Prediction:(Low)	Functional Loss	C	T	0.4284	0.0113	0.0019	4.10E-09	eBMD
rs1739842	RMVar_ID_758070	m6A	1	16016353	HSPB7	intron	m6A-Label-seq:(High)	Functional Loss	T	G	0.4100	0.0106	0.0019	1.60E-08	eBMD
rs1739842	RMVar_ID_232460	m6A	1	16016353	HSPB7	intron	m6A-Label-seq:(High)	Functional Loss	T	G	0.4100	0.0106	0.0019	1.60E-08	eBMD
rs3738097	RMVar_ID_758148	m6A	1	21568323	ALPL	intron	m6A-Label-seq:(High)	Functional Loss	T	C	0.8870	0.0188	0.0029	7.60E-10	eBMD
rs3738097	RMVar_ID_234240	m6A	1	21568323	ALPL	intron	m6A-Label-seq:(High)	Functional Loss	T	C	0.8870	0.0188	0.0029	7.60E-10	eBMD
rs3200255	RMVar_ID_63734	m1A	1	21573678	ALPL	CDS	m1A-quant-seq:(High)	Functional Loss	A	G	0.8819	0.0171	0.0028	1.40E-08	eBMD
rs3200255	RMVar_ID_63733	m1A	1	21573678	ALPL	CDS	m1A-quant-seq:(High)	Functional Loss	A	G	0.8819	0.0171	0.0028	1.40E-08	eBMD
rs3200255	RMVar_ID_110981	m1A	1	21573678	ALPL	CDS	m1A-quant-seq:(High)	Functional Loss	A	G	0.8819	0.0171	0.0028	1.40E-08	eBMD
rs3820687	RMVar_ID_63784	m1A	1	22030736	LINC00339	exon	m1A-quant-seq:(High)	Functional Loss	A	T	0.7763	0.0275	0.0022	6.10E-30	eBMD
rs2056975	RMVar_ID_9388	A-to-I	1	22082034	CDC42	intron	RNA-Seq:(High)	Functional Loss	A	G	0.0678	0.0381	0.0036	2.50E-19	eBMD
rs2056975	RMVar_ID_9389	A-to-I	1	22082034	CDC42	intron	RNA-Seq:(High)	Functional Loss	A	G	0.0678	0.0381	0.0036	2.50E-19	eBMD
rs2056975	RMVar_ID_52301	A-to-I	1	22082034	CDC42	intron	RNA-Seq:(High)	Functional Loss	A	G	0.0678	0.0381	0.0036	2.50E-19	eBMD
rs7536301	RMVar_ID_234383	m6A	1	22128486	WNT4	intron	m6A-Label-seq:(High)	Functional Loss	T	C	0.0792	0.0393	0.0034	1.50E-22	eBMD
rs7536301	RMVar_ID_234382	m6A	1	22128486	WNT4	intron	m6A-Label-seq:(High)	Functional Loss	T	C	0.0792	0.0393	0.0034	1.50E-22	eBMD
rs7536301	RMVar_ID_757882	m6A	1	22128486	WNT4	intron	m6A-Label-seq:(High)	Functional Loss	T	C	0.0792	0.0393	0.0034	1.50E-22	eBMD
rs760093	RMVar_ID_236350	m6A	1	26854937	ZDHHC18	3_UTR	MeRIP-seq:(Medium)	Functional Loss	G	A	0.7597	0.0134	0.0021	1.30E-08	eBMD
rs3010110	RMVar_ID_754898	m6A	1	26952031	KDF1	CDS	miCLIP:(High)	Functional Loss	G	A	0.7390	0.0136	0.0021	9.30E-10	eBMD
rs3010110	RMVar_ID_236491	m6A	1	26952031	KDF1	CDS	miCLIP:(High)	Functional Loss	G	A	0.7390	0.0136	0.0021	9.30E-10	eBMD
rs11209342	RMVar_ID_1188132	m6A	1	41003994	CTPS1	intron	Prediction:(Low)	Functional Loss	A	C	0.5644	0.0118	0.0018	1.80E-09	eBMD
rs11555079	RMVar_ID_1188212	m6A	1	41028081	SCMH1	3_UTR	Prediction:(Low)	Functional Gain	G	A	0.7802	0.0182	0.0022	5.10E-13	eBMD

rs10489522	RMVar_ID_499449	m6A	1	41038770	SCMH1	intron	m6A-Label-seq:(High)	Functional Loss	T	A	0.2205	-0.0182	0.0022	6.80E-13	eBMD
rs213733	RMVar_ID_756996	m6A	1	41111317	SCMH1	intron	m6A-Label-seq:(High)	Functional Loss	T	C	0.4365	-0.0119	0.0018	1.20E-09	eBMD
rs213733	RMVar_ID_499459	m6A	1	41111317	SCMH1	intron	m6A-Label-seq:(High)	Functional Loss	T	C	0.4365	-0.0119	0.0018	1.20E-09	eBMD
rs12125555	RMVar_ID_110877	m1A	1	41241439	SCMH1	intron	MeRIP-seq:(Medium)	Functional Loss	A	G	0.5634	0.0121	0.0018	9.70E-10	eBMD
rs12125555	RMVar_ID_88490	m1A	1	41241439	SCMH1	intron	MeRIP-seq:(Medium)	Functional Loss	A	G	0.5634	0.0121	0.0018	9.70E-10	eBMD
rs12044635	RMVar_ID_278687	m6A	1	68189093	WLS	intron	m6A-Label-seq:(High)	Functional Loss	T	G	0.8954	-0.0377	0.0030	1.20E-28	eBMD
rs12044635	RMVar_ID_755584	m6A	1	68189093	WLS	intron	m6A-Label-seq:(High)	Functional Loss	T	G	0.8954	-0.0377	0.0030	1.20E-28	eBMD
rs430600	RMVar_ID_1196572	m6A	1	88760316	PKN2	CDS	Prediction:(Low)	Functional Gain	T	C	0.3846	0.0187	0.0019	2.60E-15	eBMD
rs6673179	RMVar_ID_261256	m6A	1	88950833	KYAT3	intron	m6A-Label-seq:(High)	Functional Loss	T	C	0.4988	-0.0139	0.0018	2.20E-09	eBMD
rs10922710	RMVar_ID_1196902	m6A	1	89762147	LOC10192795	exon	Prediction:(Low)	Functional Gain	T	C	0.7857	0.0124	0.0022	2.80E-09	eBMD
rs6603979	RMVar_ID_636642	m6A	1	92513797	EVI5	CDS	MeRIP-seq:(Medium)	Functional Loss	A	G	0.2068	0.0136	0.0023	5.30E-10	eBMD
rs6603979	RMVar_ID_748158	m6A	1	92513797	EVI5	CDS	MeRIP-seq:(Medium)	Functional Loss	A	G	0.2068	0.0136	0.0023	5.30E-10	eBMD
rs910697	RMVar_ID_314267	m6A	1	112520504	WNT2B	CDS	MeRIP-seq:(Medium)	Functional Loss	A	G	0.5372	-0.0141	0.0018	2.70E-13	eBMD
rs6682737	RMVar_ID_314269	m6A	1	112593607	ST7L	intron	m6A-Label-seq:(High)	Functional Loss	T	C	0.5260	-0.0147	0.0018	1.10E-13	eBMD
rs2974935	RMVar_ID_1156287	m6A	1	155212058	MTX1	intron	Prediction:(Low)	Functional Loss	G	T	0.4918	0.0114	0.0018	3.90E-10	eBMD
rs2230471	RMVar_ID_752381	m6A	1	172442356	C1orf105	intron	MeRIP-seq:(Medium)	Functional Loss	A	G	0.1741	-0.0181	0.0024	1.00E-10	eBMD
rs2230471	RMVar_ID_644509	m6A	1	172442356	C1orf105	intron	MeRIP-seq:(Medium)	Functional Loss	A	G	0.1741	-0.0181	0.0024	1.00E-10	eBMD
rs4511180	RMVar_ID_1174097	m6A	1	202180311	PTPRVP	exon	Prediction:(Low)	Functional Loss	A	G	0.4072	-0.0112	0.0019	5.40E-10	eBMD
rs2057044	RMVar_ID_1177106	m6A	1	210304207	RNASEH1P3	exon	Prediction:(Low)	Functional Loss	A	T	0.8320	-0.0250	0.0025	3.90E-19	eBMD
rs4333855	RMVar_ID_661468	m6A	1	219308124	LYPLAL1	intron	m6A-Label-seq:(High)	Functional Loss	T	C	0.6980	0.0184	0.0020	2.00E-13	eBMD
rs4333855	RMVar_ID_753810	m6A	1	219308124	LYPLAL1	intron	m6A-Label-seq:(High)	Functional Loss	T	C	0.6980	0.0184	0.0020	2.00E-13	eBMD
rs1061160	RMVar_ID_661525	m6A	1	219981425	EPRS	CDS	DART-seq:(High)	Functional Loss	C	T	0.6516	0.0151	0.0019	4.80E-13	eBMD
rs1061160	RMVar_ID_757929	m6A	1	219981425	EPRS	CDS	DART-seq:(High)	Functional Loss	C	T	0.6516	0.0151	0.0019	4.80E-13	eBMD
rs12120116	RMVar_ID_754643	m6A	1	221307416	LOC10537293	exon	m6A-Label-seq:(High)	Functional Loss	A	T	0.4791	0.0173	0.0018	1.10E-13	eBMD
rs12120116	RMVar_ID_661810	m6A	1	221307416	LOC10537293	exon	m6A-Label-seq:(High)	Functional Loss	A	T	0.4791	0.0173	0.0018	1.10E-13	eBMD
rs12758915	RMVar_ID_86097	m1A	1	226870643	PSEN2	intron	m1A-IP-seq:(High)	Functional Loss	A	G	0.5176	0.0141	0.0018	1.60E-12	eBMD
rs12758915	RMVar_ID_86098	m1A	1	226870643	PSEN2	intron	m1A-IP-seq:(High)	Functional Loss	A	G	0.5176	0.0141	0.0018	1.60E-12	eBMD
rs12758915	RMVar_ID_110554	m1A	1	226870643	PSEN2	intron	m1A-IP-seq:(High)	Functional Loss	A	G	0.5176	0.0141	0.0018	1.60E-12	eBMD
rs2295994	RMVar_ID_1180152	m6A	1	227735215	JMJD4	CDS	Prediction:(Low)	Functional Loss	A	C	0.5836	0.0109	0.0019	2.70E-09	eBMD
rs1591529	RMVar_ID_589895	m6A	1	240374067	FMN2	intron	m6A-Label-seq:(High)	Functional Loss	A	T	0.5530	-0.0127	0.0018	2.90E-09	eBMD
rs1591529	RMVar_ID_756611	m6A	1	240374067	FMN2	intron	m6A-Label-seq:(High)	Functional Loss	A	T	0.5530	-0.0127	0.0018	2.90E-09	eBMD
rs15380	RMVar_ID_389148	m6A	2	10057143	CYS1	3_UTR	m6A-Label-seq:(High)	Functional Loss	T	A	0.2066	-0.0171	0.0023	2.30E-10	eBMD
rs4233729	RMVar_ID_503463	m6A	2	28869813	TRMT61B	CDS	miCLIP:(High)	Functional Loss	T	C	0.5727	-0.0149	0.0018	1.70E-12	eBMD
rs4233729	RMVar_ID_810740	m6A	2	28869813	TRMT61B	CDS	miCLIP:(High)	Functional Loss	T	C	0.5727	-0.0149	0.0018	1.70E-12	eBMD
rs72800775	RMVar_ID_735343	m6A	2	54152457	ACYP2	intron	m6A-Label-seq:(High)	Functional Loss	T	A	0.8613	0.0204	0.0026	7.90E-11	eBMD

rs72800775	RMVar_ID_815404	m6A	2	54152457	ACYP2	intron	m6A-Label-seq:(High)	Functional Loss	T	A	0.8613	0.0204	0.0026	7.90E-11	eBMD
rs2229503	RMVar_ID_735442	m6A	2	54631526	SPTBN1	CDS	MeRIP-seq:(Medium)	Functional Loss	C	T	0.8116	-0.0395	0.0023	1.70E-50	eBMD
rs2229503	RMVar_ID_818132	m6A	2	54631526	SPTBN1	CDS	MeRIP-seq:(Medium)	Functional Loss	C	T	0.8116	-0.0395	0.0023	1.70E-50	eBMD
rs2229503	RMVar_ID_142464	m5C	2	54631527	SPTBN1	CDS	BS-Seq:(High)	Functional Loss	C	T	0.8116	-0.0395	0.0023	1.70E-50	eBMD
rs2229503	RMVar_ID_139877	m5C	2	54631527	SPTBN1	CDS	BS-Seq:(High)	Functional Loss	C	T	0.8116	-0.0395	0.0023	1.70E-50	eBMD
rs145950248	RMVar_ID_735705	m6A	2	55223498	CLHC1	intron	MeRIP-seq:(Medium)	Functional Loss	C	G	0.9733	0.0397	0.0058	2.30E-10	eBMD
rs61734176	RMVar_ID_1235219	m6A	2	118842811	EN1	3_UTR	Prediction:(Low)	Functional Loss	C	A	0.8749	0.0212	0.0028	6.40E-12	eBMD
rs12988603	RMVar_ID_1240519	m6A	2	159178671	TANC1	CDS	Prediction:(Low)	Functional Loss	C	T	0.6275	-0.0188	0.0019	2.50E-17	eBMD
rs12613220	RMVar_ID_603387	m6A	2	175928077	LNPK	3_UTR	m6A-Label-seq:(High)	Functional Loss	T	A	0.9236	0.0199	0.0034	4.30E-08	eBMD
rs62187004	RMVar_ID_603416	m6A	2	175957517	LNPK	intron	m6A-Label-seq:(High)	Functional Loss	A	G	0.9235	0.0200	0.0034	3.40E-08	eBMD
rs711814	RMVar_ID_1246979	m6A	2	176104599	LOC10798580	DOWN	Prediction:(Low)	Functional Gain	T	C	0.6686	0.0139	0.0019	6.40E-13	eBMD
rs13032688	RMVar_ID_194493	m6A	2	181208637	LINC01934	exon	m6A-Label-seq:(High)	Functional Loss	T	C	0.2506	0.0178	0.0021	2.70E-15	eBMD
rs13032688	RMVar_ID_194494	m6A	2	181208637	LINC01934	exon	m6A-Label-seq:(High)	Functional Loss	T	C	0.2506	0.0178	0.0021	2.70E-15	eBMD
rs6734311	RMVar_ID_194496	m6A	2	181274781	LINC01934	exon	m6A-Label-seq:(High)	Functional Loss	T	A	0.3705	0.0130	0.0019	1.00E-09	eBMD
rs6734311	RMVar_ID_194497	m6A	2	181274781	LINC01934	exon	m6A-Label-seq:(High)	Functional Loss	T	A	0.3705	0.0130	0.0019	1.00E-09	eBMD
rs114693875	RMVar_ID_86405	m1A	2	199458729	SATB2	intron	m1A-quant-seq:(High)	Functional Loss	A	G	0.8312	0.0254	0.0024	1.20E-22	eBMD
rs114693875	RMVar_ID_86404	m1A	2	199458729	SATB2	intron	m1A-quant-seq:(High)	Functional Loss	A	G	0.8312	0.0254	0.0024	1.20E-22	eBMD
rs3751109	RMVar_ID_1258973	m6A	2	237518549	MLPH	CDS	Prediction:(Low)	Functional Loss	T	C	0.8268	0.0158	0.0024	1.20E-08	eBMD
rs11883500	RMVar_ID_598703	m6A	2	237525790	MLPH	CDS	MeRIP-seq:(Medium)	Functional Loss	C	T	0.8281	0.0151	0.0024	4.80E-08	eBMD
rs11883500	RMVar_ID_812042	m6A	2	237525790	MLPH	CDS	MeRIP-seq:(Medium)	Functional Loss	C	T	0.8281	0.0151	0.0024	4.80E-08	eBMD
rs11883500	RMVar_ID_598702	m6A	2	237525790	MLPH	CDS	MeRIP-seq:(Medium)	Functional Loss	C	T	0.8281	0.0151	0.0024	4.80E-08	eBMD
rs144190871	RMVar_ID_646394	m6A	3	33797386	PDCD6IP	intron	MeRIP-seq:(Medium)	Functional Loss	A	C	0.9885	0.0761	0.0089	3.80E-14	eBMD
rs807936	RMVar_ID_1300303	m6A	3	47281004	KIF9	intron	Prediction:(Low)	Functional Loss	C	T	0.4038	-0.0109	0.0020	1.90E-09	eBMD
rs807931	RMVar_ID_1300351	m6A	3	47347825	KLHL18	DOWN	Prediction:(Low)	Functional Gain	C	T	0.4041	-0.0111	0.0020	1.40E-09	eBMD
rs6442112	RMVar_ID_87095	m1A	3	48274544	ZNF589	DOWN	m1A-quant-seq:(High)	Functional Loss	A	G	0.2634	-0.0140	0.0021	9.70E-09	eBMD
rs6442112	RMVar_ID_113498	m1A	3	48274544	ZNF589	DOWN	m1A-quant-seq:(High)	Functional Loss	A	G	0.2634	-0.0140	0.0021	9.70E-09	eBMD
rs6442112	RMVar_ID_87096	m1A	3	48274544	ZNF589	DOWN	m1A-quant-seq:(High)	Functional Loss	A	G	0.2634	-0.0140	0.0021	9.70E-09	eBMD
rs9860285	RMVar_ID_484907	m6A	3	49317942	USP4	intron	MeRIP-seq:(Medium)	Functional Loss	G	T	0.2569	-0.0148	0.0021	5.20E-10	eBMD
rs11242	RMVar_ID_487208	m6A	3	53091908	RFT1	CDS	miCLIP:(High)	Functional Loss	T	C	0.4412	-0.0119	0.0019	2.70E-08	eBMD
rs11242	RMVar_ID_792758	m6A	3	53091908	RFT1	CDS	miCLIP:(High)	Functional Loss	T	C	0.4412	-0.0119	0.0019	2.70E-08	eBMD
rs73086405	RMVar_ID_487628	m6A	3	56272034	ERC2	intron	m6A-Label-seq:(High)	Functional Loss	T	A	0.6119	0.0186	0.0019	9.90E-18	eBMD
rs12629096	RMVar_ID_487630	m6A	3	56275606	ERC2	intron	m6A-Label-seq:(High)	Functional Loss	A	C	0.6113	0.0187	0.0019	5.50E-18	eBMD
rs7432266	RMVar_ID_29890	A-to-I	3	57688184	DENND6A	intron	RNA-Seq:(High)	Functional Loss	T	C	0.1779	-0.0178	0.0024	6.10E-10	eBMD
rs7432266	RMVar_ID_29889	A-to-I	3	57688184	DENND6A	intron	RNA-Seq:(High)	Functional Loss	T	C	0.1779	-0.0178	0.0024	6.10E-10	eBMD
rs513154	RMVar_ID_685757	m6A	3	101234368	IMPG2	intron	m6A-Label-seq:(High)	Functional Loss	T	G	0.3428	-0.0120	0.0019	1.70E-09	eBMD

rs513154	RMVar_ID_685758	m6A	3	101234368	IMPG2	intron	m6A-Label-seq:(High)	Functional Loss	T	G	0.3428	-0.0120	0.0019	1.70E-09	eBMD
rs513154	RMVar_ID_685756	m6A	3	101234368	IMPG2	intron	m6A-Label-seq:(High)	Functional Loss	T	G	0.3428	-0.0120	0.0019	1.70E-09	eBMD
rs4679245	RMVar_ID_41376	A-to-I	3	126465607	ZXDC	intron	RNA-Seq:(High)	Functional Loss	T	C	0.3056	-0.0113	0.0020	2.50E-09	eBMD
rs10935464	RMVar_ID_695038	m6A	3	142475914	ATR	intron	MeRIP-seq:(Medium)	Functional Loss	G	C	0.8403	0.0165	0.0025	2.40E-08	eBMD
rs10935464	RMVar_ID_796033	m6A	3	142475914	ATR	intron	MeRIP-seq:(Medium)	Functional Loss	G	C	0.8403	0.0165	0.0025	2.40E-08	eBMD
rs10935464	RMVar_ID_695037	m6A	3	142475914	ATR	intron	MeRIP-seq:(Medium)	Functional Loss	G	C	0.8403	0.0165	0.0025	2.40E-08	eBMD
rs75072999	RMVar_ID_1309574	m6A	4	850995	GAK	CDS	Prediction:(Low)	Functional Gain	G	A	0.9049	-0.0301	0.0032	1.80E-20	eBMD
rs6815946	RMVar_ID_1309772	m6A	4	1001501	IDUA	CDS	Prediction:(Low)	Functional Gain	T	C	0.8485	0.0675	0.0026	2.50E-116	eBMD
rs6815946	RMVar_ID_1309772	m6A	4	1001501	IDUA	CDS	Prediction:(Low)	Functional Gain	T	C	0.8485	-0.0607	0.0091	3.80E-11	Fracture
rs115790973	RMVar_ID_1309776	m6A	4	1002770	IDUA	CDS	Prediction:(Low)	Functional Gain	C	G	0.8499	0.0684	0.0026	2.90E-119	eBMD
rs115790973	RMVar_ID_1309777	m6A	4	1002770	IDUA	CDS	Prediction:(Low)	Functional Gain	C	G	0.8499	0.0684	0.0026	2.90E-119	eBMD
rs115790973	RMVar_ID_1309775	m6A	4	1002770	IDUA	CDS	Prediction:(Low)	Functional Gain	C	G	0.8499	0.0684	0.0026	2.90E-119	eBMD
rs115790973	RMVar_ID_1309777	m6A	4	1002770	IDUA	CDS	Prediction:(Low)	Functional Gain	C	G	0.8499	-0.0626	0.0092	1.00E-11	Fracture
rs115790973	RMVar_ID_1309775	m6A	4	1002770	IDUA	CDS	Prediction:(Low)	Functional Gain	C	G	0.8499	-0.0626	0.0092	1.00E-11	Fracture
rs115790973	RMVar_ID_1309776	m6A	4	1002770	IDUA	CDS	Prediction:(Low)	Functional Gain	C	G	0.8499	-0.0626	0.0092	1.00E-11	Fracture
rs3796619	RMVar_ID_1309887	m6A	4	1101518	RNF212	intron	Prediction:(Low)	Functional Gain	A	G	0.3173	0.0222	0.0020	9.80E-23	eBMD
rs11247975	RMVar_ID_126236	m5C	4	1171342	SPON2	CDS	BS-Seq:(High)	Functional Loss	G	T	0.3195	-0.0224	0.0020	1.60E-23	eBMD
rs11247975	RMVar_ID_140483	m5C	4	1171342	SPON2	CDS	BS-Seq:(High)	Functional Loss	G	T	0.3195	-0.0224	0.0020	1.60E-23	eBMD
rs11247975	RMVar_ID_126237	m5C	4	1171342	SPON2	CDS	BS-Seq:(High)	Functional Loss	G	T	0.3195	-0.0224	0.0020	1.60E-23	eBMD
rs11538062	RMVar_ID_762837	m6A	4	1171975	SPON2	CDS	MeRIP-seq:(Medium)	Functional Loss	C	T	0.8608	0.0327	0.0027	1.60E-28	eBMD
rs11538062	RMVar_ID_373288	m6A	4	1171975	SPON2	CDS	MeRIP-seq:(Medium)	Functional Loss	C	T	0.8608	0.0327	0.0027	1.60E-28	eBMD
rs6838561	RMVar_ID_373641	m6A	4	1386245	UVSSA	3_UTR	MeRIP-seq:(Medium)	Functional Loss	A	G	0.3182	0.0172	0.0020	1.80E-16	eBMD
rs901705	RMVar_ID_1325029	m6A	4	38366983	LINC02513	exon	Prediction:(Low)	Functional Gain	T	G	0.3194	0.0146	0.0020	1.10E-10	eBMD
rs901705	RMVar_ID_1325028	m6A	4	38366983	LINC02513	exon	Prediction:(Low)	Functional Gain	T	G	0.3194	0.0146	0.0020	1.10E-10	eBMD
rs17500058	RMVar_ID_153736	m6A	4	38512672	LINC01259	exon	m6A-Label-seq:(High)	Functional Loss	A	G	0.7527	-0.0179	0.0022	7.20E-15	eBMD
rs112068329	RMVar_ID_1314207	m6A	4	105369330	PPA2	3_UTR	Prediction:(Low)	Functional Loss	T	G	0.8683	0.0172	0.0027	1.20E-08	eBMD
rs8733	RMVar_ID_58373	m1A	4	165342967	MSMO1	3_UTR	m1A-quant-seq:(High)	Functional Loss	A	G	0.7626	-0.0157	0.0022	2.40E-11	eBMD
rs8733	RMVar_ID_111207	m1A	4	165342967	MSMO1	3_UTR	m1A-quant-seq:(High)	Functional Loss	A	G	0.7626	-0.0157	0.0022	2.40E-11	eBMD
rs8733	RMVar_ID_58374	m1A	4	165342967	MSMO1	3_UTR	m1A-quant-seq:(High)	Functional Loss	A	G	0.7626	-0.0157	0.0022	2.40E-11	eBMD
rs676067	RMVar_ID_1354392	m6A	5	41951507	FBXO4	intron	Prediction:(Low)	Functional Loss	G	A	0.7561	-0.0154	0.0022	6.30E-11	eBMD
rs6875903	RMVar_ID_440797	m6A	5	55476166	PPAP2A	intron	m6A-Label-seq:(High)	Functional Loss	T	G	0.8310	-0.0182	0.0025	4.70E-11	eBMD
rs6875903	RMVar_ID_820629	m6A	5	55476166	PPAP2A	intron	m6A-Label-seq:(High)	Functional Loss	T	G	0.8310	-0.0182	0.0025	4.70E-11	eBMD
rs6556919	RMVar_ID_254016	m6A	5	96364161	LOC10192971	exon	m6A-Label-seq:(High)	Functional Loss	A	C	0.3311	-0.0148	0.0020	7.90E-14	eBMD
rs1045706	RMVar_ID_824484	m6A	5	109378599	PJA2	CDS	MeRIP-seq:(Medium)	Functional Loss	T	C	0.4062	-0.0145	0.0019	4.80E-11	eBMD
rs1045706	RMVar_ID_391374	m6A	5	109378599	PJA2	CDS	MeRIP-seq:(Medium)	Functional Loss	T	C	0.4062	-0.0145	0.0019	4.80E-11	eBMD

rs11241184	RMVar_ID_391646	m6A	5	112762060	APC	intron	m6A-Label-seq:(High)	Functional Loss	A	G	0.4882	0.0137	0.0018	2.10E-12	eBMD
rs390092	RMVar_ID_391651	m6A	5	112810500	APC	intron	m6A-Label-seq:(High)	Functional Loss	T	G	0.5058	0.0142	0.0018	5.10E-13	eBMD
rs6595440	RMVar_ID_1337638	m6A	5	123383045	CEP120	CDS	Prediction:(Low)	Functional Gain	G	C	0.5645	-0.0113	0.0019	8.20E-11	eBMD
rs11958836	RMVar_ID_686549	m6A	5	123402629	CEP120	intron	MeRIP-seq:(Medium)	Functional Loss	G	A	0.5662	-0.0113	0.0019	9.80E-11	eBMD
rs11958836	RMVar_ID_686550	m6A	5	123402629	CEP120	intron	MeRIP-seq:(Medium)	Functional Loss	G	A	0.5662	-0.0113	0.0019	9.80E-11	eBMD
rs7732639	RMVar_ID_114877	m1A	5	128537912	FBN2	5_UTR	m1A-quant-seq:(High)	Functional Loss	T	C	0.6840	0.0146	0.0020	1.70E-10	eBMD
rs7732639	RMVar_ID_105215	m1A	5	128537912	FBN2	5_UTR	m1A-quant-seq:(High)	Functional Loss	T	C	0.6840	0.0146	0.0020	1.70E-10	eBMD
rs7732639	RMVar_ID_105214	m1A	5	128537912	FBN2	5_UTR	m1A-quant-seq:(High)	Functional Loss	T	C	0.6840	0.0146	0.0020	1.70E-10	eBMD
rs30177	RMVar_ID_820778	m6A	5	132866717	UQCRQ	intron	MeRIP-seq:(Medium)	Functional Loss	C	G	0.2551	0.0148	0.0021	8.10E-09	eBMD
rs30177	RMVar_ID_687786	m6A	5	132866717	UQCRQ	intron	MeRIP-seq:(Medium)	Functional Loss	C	G	0.2551	0.0148	0.0021	8.10E-09	eBMD
rs6906505	RMVar_ID_1362266	m6A	6	7213016	RREB1	intron	Prediction:(Low)	Functional Loss	C	T	0.5214	-0.0136	0.0018	5.70E-10	eBMD
rs2073531	RMVar_ID_1373098	m6A	6	26375032	BTN3A2	intron	Prediction:(Low)	Functional Loss	A	G	0.8676	-0.0171	0.0027	1.10E-10	eBMD
rs3757138	RMVar_ID_285949	m6A	6	26375875	BTN3A2	3_UTR	miCLIP:(High)	Functional Loss	A	G	0.8673	-0.0170	0.0027	1.30E-10	eBMD
rs3757138	RMVar_ID_809012	m6A	6	26375875	BTN3A2	3_UTR	miCLIP:(High)	Functional Loss	A	G	0.8673	-0.0170	0.0027	1.30E-10	eBMD
rs56405707	RMVar_ID_144848	m5U	6	27672469	TRS-CGA3-1	exon	FICC-Seq&miCLIP:(High	Functional Loss	G	A	0.8820	-0.0194	0.0028	1.80E-11	eBMD
rs9986596	RMVar_ID_123397	m5C	6	28251883	ZKSCAN4	CDS	BS-Seq:(High)	Functional Loss	G	A	0.8375	-0.0145	0.0025	9.60E-09	eBMD
rs9986596	RMVar_ID_142232	m5C	6	28251883	ZKSCAN4	CDS	BS-Seq:(High)	Functional Loss	G	A	0.8375	-0.0145	0.0025	9.60E-09	eBMD
rs9986596	RMVar_ID_123398	m5C	6	28251883	ZKSCAN4	CDS	BS-Seq:(High)	Functional Loss	G	A	0.8375	-0.0145	0.0025	9.60E-09	eBMD
rs853678	RMVar_ID_287202	m6A	6	28329536	ZSCAN31	CDS	MeRIP-seq:(Medium)	Functional Loss	T	A	0.8355	-0.0142	0.0025	3.70E-09	eBMD
rs853678	RMVar_ID_806285	m6A	6	28329536	ZSCAN31	CDS	MeRIP-seq:(Medium)	Functional Loss	T	A	0.8355	-0.0142	0.0025	3.70E-09	eBMD
rs853678	RMVar_ID_287201	m6A	6	28329536	ZSCAN31	CDS	MeRIP-seq:(Medium)	Functional Loss	T	A	0.8355	-0.0142	0.0025	3.70E-09	eBMD
rs9260119	RMVar_ID_1374301	m6A	6	29942440	HLA-A	UP	Prediction:(Low)	Functional Gain	A	T	0.4234	0.0195	0.0019	1.60E-22	eBMD
rs9260119	RMVar_ID_1374302	m6A	6	29942440	HLA-A	UP	Prediction:(Low)	Functional Gain	A	T	0.4234	0.0195	0.0019	1.60E-22	eBMD
rs9260146	RMVar_ID_1374312	m6A	6	29943131	HLA-A	intron	Prediction:(Low)	Functional Gain	C	T	0.8043	-0.0221	0.0023	1.80E-19	eBMD
rs1061815	RMVar_ID_287800	m6A	6	29945596	HLA-A	3_UTR	MeRIP-seq:(Medium)	Functional Loss	C	G	0.8044	-0.0221	0.0023	2.10E-19	eBMD
rs1061815	RMVar_ID_287799	m6A	6	29945596	HLA-A	3_UTR	MeRIP-seq:(Medium)	Functional Loss	C	G	0.8044	-0.0221	0.0023	2.10E-19	eBMD
rs35835721	RMVar_ID_287831	m6A	6	29974607	MICD	exon	m6A-Label-seq:(High)	Functional Loss	T	G	0.7139	0.0125	0.0020	4.20E-09	eBMD
rs35835721	RMVar_ID_809663	m6A	6	29974607	MICD	exon	m6A-Label-seq:(High)	Functional Loss	T	G	0.7139	0.0125	0.0020	4.20E-09	eBMD
rs36019691	RMVar_ID_805118	m6A	6	29974607	MICD	exon	m6A-Label-seq:(High)	Functional Loss	T	C	0.7139	0.0125	0.0020	4.20E-09	eBMD
rs36019691	RMVar_ID_287829	m6A	6	29974607	MICD	exon	m6A-Label-seq:(High)	Functional Loss	T	C	0.7139	0.0125	0.0020	4.20E-09	eBMD
rs2285800	RMVar_ID_361152	m6A	6	30289719	HCG18	3_UTR	MeRIP-seq:(Medium)	Functional Loss	C	G	0.7353	0.0127	0.0021	6.70E-09	eBMD
rs2285800	RMVar_ID_806384	m6A	6	30289719	HCG18	3_UTR	MeRIP-seq:(Medium)	Functional Loss	C	G	0.7353	0.0127	0.0021	6.70E-09	eBMD
rs186215159	RMVar_ID_1380053	m6A	6	35090322	ANKS1A	3_UTR	Prediction:(Low)	Functional Loss	G	A	0.9811	0.0410	0.0067	2.80E-10	eBMD
rs12206659	RMVar_ID_541055	m6A	6	54004423	MLIP-IT1	intron	m6A-Label-seq:(High)	Functional Loss	T	C	0.5725	0.0122	0.0018	8.00E-10	eBMD
rs4715631	RMVar_ID_804448	m6A	6	56552747	DST	CDS	MeRIP-seq:(Medium)	Functional Loss	T	C	0.2884	-0.0118	0.0020	1.10E-08	eBMD

rs4715631	RMVar_ID_541276	m6A	6	56552747	DST	CDS	MeRIP-seq:(Medium)	Functional Loss	T	C	0.2884	-0.0118	0.0020	1.10E-08	eBMD
rs45477497	RMVar_ID_541751	m6A	6	57103511	ZNF451	intron	MeRIP-seq:(Medium)	Functional Loss	G	T	0.9452	0.0257	0.0040	1.40E-08	eBMD
rs12660627	RMVar_ID_507256	m6A	6	73794501	CD109	intron	m6A-Label-seq:(High)	Functional Loss	A	G	0.4848	0.0233	0.0018	5.30E-31	eBMD
rs293526	RMVar_ID_1387238	m6A	6	82938365	UBE3D	intron	Prediction:(Low)	Functional Loss	C	T	0.2926	0.0216	0.0020	3.00E-19	eBMD
rs12209871	RMVar_ID_1387246	m6A	6	83036347	UBE3D	intron	Prediction:(Low)	Functional Gain	A	G	0.8546	-0.0194	0.0026	3.00E-10	eBMD
rs6902288	RMVar_ID_327995	m6A	6	127447618	KIAA0408	CDS	MeRIP-seq:(Medium)	Functional Loss	G	A	0.3963	-0.0157	0.0019	1.00E-12	eBMD
rs6902288	RMVar_ID_327994	m6A	6	127447618	KIAA0408	CDS	MeRIP-seq:(Medium)	Functional Loss	G	A	0.3963	-0.0157	0.0019	1.00E-12	eBMD
rs6902288	RMVar_ID_804754	m6A	6	127447618	KIAA0408	CDS	MeRIP-seq:(Medium)	Functional Loss	G	A	0.3963	-0.0157	0.0019	1.00E-12	eBMD
rs6975	RMVar_ID_1366544	m6A	6	130834964	SMLR1	3_UTR	Prediction:(Low)	Functional Loss	G	T	0.7379	-0.0188	0.0021	5.70E-13	eBMD
rs6975	RMVar_ID_1366543	m6A	6	130834964	SMLR1	3_UTR	Prediction:(Low)	Functional Loss	G	T	0.7379	-0.0188	0.0021	5.70E-13	eBMD
rs3823310	RMVar_ID_809307	m6A	6	151353191	AKAP12	CDS	MeRIP-seq:(Medium)	Functional Loss	A	C	0.3268	-0.0166	0.0020	8.20E-15	eBMD
rs3823310	RMVar_ID_663132	m6A	6	151353191	AKAP12	CDS	MeRIP-seq:(Medium)	Functional Loss	A	C	0.3268	-0.0166	0.0020	8.20E-15	eBMD
rs9397054	RMVar_ID_1369505	m6A	6	151468793	ARMT1	CDS	Prediction:(Low)	Functional Loss	C	T	0.8973	0.0474	0.0030	2.80E-44	eBMD
rs9383935	RMVar_ID_1369539	m6A	6	151618697	CCDC170	3_UTR	Prediction:(Low)	Functional Loss	C	T	0.9220	0.0427	0.0034	1.30E-27	eBMD
rs7740449	RMVar_ID_663340	m6A	6	152253365	SYNE1	intron	m6A-Label-seq:(High)	Functional Loss	T	C	0.3837	0.0167	0.0019	1.20E-15	eBMD
rs668261	RMVar_ID_664158	m6A	6	158463191	TULP4	intron	m6A-Label-seq:(High)	Functional Loss	T	A	0.6655	-0.0128	0.0019	4.60E-11	eBMD
rs668261	RMVar_ID_806491	m6A	6	158463191	TULP4	intron	m6A-Label-seq:(High)	Functional Loss	T	A	0.6655	-0.0128	0.0019	4.60E-11	eBMD
rs11975534	RMVar_ID_510509	m6A	7	15686504	MEOX2	5_UTR	MeRIP-seq:(Medium)	Functional Loss	A	C	0.3676	-0.0290	0.0019	2.60E-46	eBMD
rs11975534	RMVar_ID_510510	m6A	7	15686504	MEOX2	5_UTR	MeRIP-seq:(Medium)	Functional Loss	A	C	0.3676	-0.0290	0.0019	2.60E-46	eBMD
rs11975534	RMVar_ID_828358	m6A	7	15686504	MEOX2	5_UTR	MeRIP-seq:(Medium)	Functional Loss	A	C	0.3676	-0.0290	0.0019	2.60E-46	eBMD
rs1121731	RMVar_ID_510808	m6A	7	19582372	LOC10537518	exon	m6A-Label-seq:(High)	Functional Loss	A	C	0.6268	-0.0128	0.0019	1.30E-10	eBMD
rs6461998	RMVar_ID_512406	m6A	7	27241538	EVX1-AS	intron	m6A-Label-seq:(High)	Functional Loss	T	C	0.0559	0.0375	0.0040	6.50E-18	eBMD
rs7795470	RMVar_ID_512411	m6A	7	27246764	EVX1	3_UTR	MeRIP-seq:(Medium)	Functional Loss	T	C	0.0202	0.0387	0.0065	7.70E-09	eBMD
rs7795470	RMVar_ID_512412	m6A	7	27246764	EVX1	3_UTR	MeRIP-seq:(Medium)	Functional Loss	T	C	0.0202	0.0387	0.0065	7.70E-09	eBMD
rs7795470	RMVar_ID_828393	m6A	7	27246764	EVX1	3_UTR	MeRIP-seq:(Medium)	Functional Loss	T	C	0.0202	0.0387	0.0065	7.70E-09	eBMD
rs7795470	RMVar_ID_512410	m6A	7	27246764	EVX1	3_UTR	MeRIP-seq:(Medium)	Functional Loss	T	C	0.0202	0.0387	0.0065	7.70E-09	eBMD
rs2722276	RMVar_ID_580807	m6A	7	37949577	EPDR1	intron	MeRIP-seq:(Medium)	Functional Loss	G	A	0.4731	0.0164	0.0018	1.30E-18	eBMD
rs117893851	RMVar_ID_1415575	m6A	7	92471521	ERVW-1	intron	Prediction:(Low)	Functional Loss	G	A	0.9212	0.0205	0.0034	2.10E-08	eBMD
rs62470375	RMVar_ID_349640	m6A	7	96664376	SEM1	intron	MeRIP-seq:(Medium)	Functional Loss	A	G	0.8929	-0.0203	0.0029	8.80E-10	eBMD
rs7787376	RMVar_ID_831199	m6A	7	97116762	ACN9	5_UTR	m6A-Label-seq:(High)	Functional Loss	A	G	0.6575	0.0176	0.0019	1.20E-15	eBMD
rs7787376	RMVar_ID_349707	m6A	7	97116762	ACN9	5_UTR	m6A-Label-seq:(High)	Functional Loss	A	G	0.6575	0.0176	0.0019	1.20E-15	eBMD
rs28680963	RMVar_ID_1417519	m6A	7	100307836	SPDYE3	5_UTR	Prediction:(Low)	Functional Loss	C	G	0.8109	0.0147	0.0023	5.20E-10	eBMD
rs7792525	RMVar_ID_351718	m6A	7	100374497	PILRA	intron	MeRIP-seq:(Medium)	Functional Loss	A	G	0.8112	0.0146	0.0023	8.30E-10	eBMD
rs7792525	RMVar_ID_829541	m6A	7	100374497	PILRA	intron	MeRIP-seq:(Medium)	Functional Loss	A	G	0.8112	0.0146	0.0023	8.30E-10	eBMD
rs6962151	RMVar_ID_115132	m1A	7	100430861	MEPCE	CDS	MeRIP-seq:(Medium)	Functional Loss	T	C	0.8041	0.0147	0.0023	2.70E-10	eBMD

rs6962151	RMVar_ID_74483	m1A	7	100430861	MEPCE	CDS	MeRIP-seq:(Medium)	Functional Loss	T	C	0.8041	0.0147	0.0023	2.70E-10	eBMD
rs6962151	RMVar_ID_351769	m6A	7	100430861	MEPCE	CDS	MeRIP-seq:(Medium)	Functional Loss	T	C	0.8041	0.0147	0.0023	2.70E-10	eBMD
rs6962151	RMVar_ID_829742	m6A	7	100430861	MEPCE	CDS	MeRIP-seq:(Medium)	Functional Loss	T	C	0.8041	0.0147	0.0023	2.70E-10	eBMD
rs11559028	RMVar_ID_352025	m6A	7	100707240	POP7	CDS	MeRIP-seq:(Medium)	Functional Loss	C	A	0.9605	-0.0291	0.0047	3.00E-09	eBMD
rs11559028	RMVar_ID_352026	m6A	7	100707240	POP7	CDS	MeRIP-seq:(Medium)	Functional Loss	C	A	0.9605	-0.0291	0.0047	3.00E-09	eBMD
rs79163323	RMVar_ID_1418011	m6A	7	100748415	ZAN	CDS	Prediction:(Low)	Functional Loss	C	T	0.9656	-0.0325	0.0050	5.90E-09	eBMD
rs61729506	RMVar_ID_322948	m6A	7	149266560	ZNF783	CDS	MeRIP-seq:(Medium)	Functional Loss	C	T	0.9320	0.0222	0.0036	1.60E-09	eBMD
rs112796174	RMVar_ID_1562955	m7G	7	149289177	LOC155060	exon	MeRIP-seq:(Medium)	Functional Loss	G	T	0.9319	0.0223	0.0036	1.80E-09	eBMD
rs112796174	RMVar_ID_1562954	m7G	7	149289177	LOC155060	exon	MeRIP-seq:(Medium)	Functional Loss	G	T	0.9319	0.0223	0.0036	1.80E-09	eBMD
rs1050734	RMVar_ID_434132	m6A	7	151076478	SLC4A2	3_UTR	MeRIP-seq:(Medium)	Functional Loss	C	A	0.7518	0.0176	0.0021	1.40E-13	eBMD
rs897917	RMVar_ID_1401422	m6A	7	151212533	ABCF2	3_UTR	Prediction:(Low)	Functional Gain	G	C	0.5882	0.0098	0.0019	8.60E-09	eBMD
rs2608288	RMVar_ID_434227	m6A	7	151213367	ABCF2	3_UTR	MeRIP-seq:(Medium)	Functional Loss	C	G	0.4115	-0.0105	0.0019	8.40E-10	eBMD
rs2945254	RMVar_ID_654379	m6A	8	8235719	FAM86B3P	intron	MeRIP-seq:(Medium)	Functional Loss	G	T	0.8663	-0.0312	0.0028	1.60E-22	eBMD
rs13256369	RMVar_ID_654649	m6A	8	8719869	RF00017-4499	intron	m6A-Label-seq:(High)	Functional Loss	T	C	0.7450	-0.0256	0.0021	1.00E-25	eBMD
rs13256369	RMVar_ID_845065	m6A	8	8719869	RF00017-4499	intron	m6A-Label-seq:(High)	Functional Loss	T	C	0.7450	-0.0256	0.0021	1.00E-25	eBMD
rs2979247	RMVar_ID_1421525	m6A	8	9031438	ERI1	3_UTR	Prediction:(Low)	Functional Loss	A	G	0.5810	-0.0313	0.0019	3.40E-42	eBMD
rs11780338	RMVar_ID_102469	m1A	8	9032336	ERI1	3_UTR	m1A-quant-seq:(High)	Functional Loss	A	G	0.6788	0.0245	0.0020	3.20E-22	eBMD
rs11780338	RMVar_ID_102470	m1A	8	9032336	ERI1	3_UTR	m1A-quant-seq:(High)	Functional Loss	A	G	0.6788	0.0245	0.0020	3.20E-22	eBMD
rs330917	RMVar_ID_654871	m6A	8	9139685	PPP1R3B	3_UTR	MeRIP-seq:(Medium)	Functional Loss	G	T	0.7103	-0.0280	0.0020	2.30E-31	eBMD
rs330917	RMVar_ID_845792	m6A	8	9139685	PPP1R3B	3_UTR	MeRIP-seq:(Medium)	Functional Loss	G	T	0.7103	-0.0280	0.0020	2.30E-31	eBMD
rs592420	RMVar_ID_1421706	m6A	8	9902922	LINC00599	exon	Prediction:(Low)	Functional Gain	A	G	0.7522	-0.0215	0.0022	4.90E-16	eBMD
rs71516561	RMVar_ID_655017	m6A	8	10219867	MSRA	intron	m6A-Label-seq:(High)	Functional Loss	T	G	0.7485	-0.0182	0.0021	5.80E-16	eBMD
rs2952194	RMVar_ID_655022	m6A	8	10260492	MSRA	intron	MeRIP-seq:(Medium)	Functional Loss	A	G	0.8088	-0.0145	0.0024	1.40E-08	eBMD
rs2975648	RMVar_ID_655023	m6A	8	10278466	MSRA	intron	m6A-Label-seq:(High)	Functional Loss	T	C	0.5666	-0.0338	0.0019	9.50E-51	eBMD
rs6601438	RMVar_ID_655025	m6A	8	10322157	MSRA	intron	m6A-Label-seq:(High)	Functional Loss	A	C	0.2732	-0.0290	0.0021	7.50E-34	eBMD
rs55642448	RMVar_ID_1421883	m6A	8	10607241	RP1L1	CDS	Prediction:(Low)	Functional Gain	C	T	0.6134	-0.0169	0.0019	9.80E-13	eBMD
rs4841497	RMVar_ID_655209	m6A	8	11127630	XKR6	intron	m6A-Label-seq:(High)	Functional Loss	A	G	0.6152	0.0286	0.0019	2.30E-35	eBMD
rs4841497	RMVar_ID_843249	m6A	8	11127630	XKR6	intron	m6A-Label-seq:(High)	Functional Loss	A	G	0.6152	0.0286	0.0019	2.30E-35	eBMD
rs2293856	RMVar_ID_1422257	m6A	8	11319964	MTMR9	intron	Prediction:(Low)	Functional Loss	C	T	0.5977	-0.0323	0.0019	3.90E-46	eBMD
rs899368	RMVar_ID_655306	m6A	8	11643283	LOC10537924	intron	m6A-Label-seq:(High)	Functional Loss	T	A	0.1170	0.0256	0.0029	2.70E-12	eBMD
rs904018	RMVar_ID_1422499	m6A	8	11759015	GATA4	3_UTR	Prediction:(Low)	Functional Gain	T	C	0.3570	0.0190	0.0019	1.10E-22	eBMD
rs2645429	RMVar_ID_1422578	m6A	8	11802558	FDFT1	intron	Prediction:(Low)	Functional Gain	A	G	0.2435	-0.0246	0.0022	3.70E-22	eBMD
rs2645429	RMVar_ID_1422577	m6A	8	11802558	FDFT1	intron	Prediction:(Low)	Functional Gain	A	G	0.2435	-0.0246	0.0022	3.70E-22	eBMD
rs1047643	RMVar_ID_115736	m1A	8	11802853	FDFT1	CDS	MeRIP-seq:(Medium)	Functional Loss	T	C	0.8293	0.0164	0.0025	5.90E-09	eBMD
rs1047643	RMVar_ID_102493	m1A	8	11802853	FDFT1	CDS	MeRIP-seq:(Medium)	Functional Loss	T	C	0.8293	0.0164	0.0025	5.90E-09	eBMD

rs8898	RMVar_ID_655550	m6A	8	11845033	CTSB	3_UTR	miCLIP:(High)	Functional Loss	T	C	0.6533	-0.0177	0.0019	6.50E-13	eBMD
rs8898	RMVar_ID_845032	m6A	8	11845033	CTSB	3_UTR	miCLIP:(High)	Functional Loss	T	C	0.6533	-0.0177	0.0019	6.50E-13	eBMD
rs7464553	RMVar_ID_655566	m6A	8	11920392	OR7E158P	exon	m6A-Label-seq:(High)	Functional Loss	T	C	0.4472	-0.0317	0.0019	7.70E-47	eBMD
rs2709618	RMVar_ID_115733	m1A	8	25410106	DOCK5	CDS	MeRIP-seq:(Medium)	Functional Loss	A	G	0.3691	0.0140	0.0019	3.50E-15	eBMD
rs2709618	RMVar_ID_99375	m1A	8	25410106	DOCK5	CDS	MeRIP-seq:(Medium)	Functional Loss	A	G	0.3691	0.0140	0.0019	3.50E-15	eBMD
rs6468694	RMVar_ID_1442370	m6A	8	99853637	VPS13B	CDS	Prediction:(Low)	Functional Loss	G	A	0.8744	-0.0207	0.0028	3.70E-12	eBMD
rs631775	RMVar_ID_741965	m6A	8	108240517	EIF3E	intron	m6A-Label-seq:(High)	Functional Loss	T	C	0.4257	0.0134	0.0019	2.00E-09	eBMD
rs631775	RMVar_ID_846871	m6A	8	108240517	EIF3E	intron	m6A-Label-seq:(High)	Functional Loss	T	C	0.4257	0.0134	0.0019	2.00E-09	eBMD
rs2005617	RMVar_ID_865560	m6A	9	33791166	PRSS3	intron	m6A-Label-seq:(High)	Functional Loss	T	C	0.3755	-0.0128	0.0019	1.10E-10	eBMD
rs2005617	RMVar_ID_238503	m6A	9	33791166	PRSS3	intron	m6A-Label-seq:(High)	Functional Loss	T	C	0.3755	-0.0128	0.0019	1.10E-10	eBMD
rs2005617	RMVar_ID_238504	m6A	9	33791166	PRSS3	intron	m6A-Label-seq:(High)	Functional Loss	T	C	0.3755	-0.0128	0.0019	1.10E-10	eBMD
rs307658	RMVar_ID_116986	m1A	9	33941761	UBAP2	CDS	m1A-quant-seq:(High)	Functional Loss	T	C	0.6223	0.0127	0.0019	2.20E-10	eBMD
rs307658	RMVar_ID_64186	m1A	9	33941761	UBAP2	CDS	m1A-quant-seq:(High)	Functional Loss	T	C	0.6223	0.0127	0.0019	2.20E-10	eBMD
rs11141677	RMVar_ID_1465006	m6A	9	87084277	NFYCP2	exon	Prediction:(Low)	Functional Loss	C	G	0.7508	-0.0155	0.0021	2.60E-08	eBMD
rs10821035	RMVar_ID_689272	m6A	9	92873793	ZNF484	intron	m6A-Label-seq:(High)	Functional Loss	T	C	0.6359	0.0181	0.0019	5.50E-15	eBMD
rs10821035	RMVar_ID_867084	m6A	9	92873793	ZNF484	intron	m6A-Label-seq:(High)	Functional Loss	T	C	0.6359	0.0181	0.0019	5.50E-15	eBMD
rs11793637	RMVar_ID_689507	m6A	9	93653617	PHF2	intron	m6A-Label-seq:(High)	Functional Loss	A	G	0.4973	0.0162	0.0019	1.90E-15	eBMD
rs11793637	RMVar_ID_689508	m6A	9	93653617	PHF2	intron	m6A-Label-seq:(High)	Functional Loss	A	G	0.4973	0.0162	0.0019	1.90E-15	eBMD
rs3217751	RMVar_ID_865223	m6A	9	127790634	CDK9	3_UTR	miCLIP:(High)	Functional Loss	A	G	0.5876	-0.0135	0.0019	2.20E-10	eBMD
rs3217751	RMVar_ID_712249	m6A	9	127790634	CDK9	3_UTR	miCLIP:(High)	Functional Loss	A	G	0.5876	-0.0135	0.0019	2.20E-10	eBMD
rs10901225	RMVar_ID_714204	m6A	9	130605964	FUBP3	intron	m6A-Label-seq:(High)	Functional Loss	A	C	0.7494	0.0241	0.0022	2.20E-24	eBMD
rs10901225	RMVar_ID_714203	m6A	9	130605964	FUBP3	intron	m6A-Label-seq:(High)	Functional Loss	A	C	0.7494	0.0241	0.0022	2.20E-24	eBMD
rs41302673	RMVar_ID_1452935	m6A	9	133405412	STKLD1	CDS	Prediction:(Low)	Functional Loss	T	G	0.9096	0.0264	0.0032	5.00E-12	eBMD
rs739468	RMVar_ID_715939	m6A	9	133461126	CACFD1	intron	m6A-Label-seq:(High)	Functional Loss	T	G	0.1353	-0.0241	0.0027	7.30E-14	eBMD
rs739468	RMVar_ID_715938	m6A	9	133461126	CACFD1	intron	m6A-Label-seq:(High)	Functional Loss	T	G	0.1353	-0.0241	0.0027	7.30E-14	eBMD
rs1050046	RMVar_ID_869503	m6A	10	3779328	KLF6	3_UTR	Prediction:(Low)	Functional Loss	G	A	0.9210	0.0224	0.0034	4.70E-09	eBMD
rs2461940	RMVar_ID_551992	m6A	10	20116369	PLXDC2	intron	m6A-Label-seq:(High)	Functional Loss	T	C	0.5203	0.0183	0.0018	4.60E-16	eBMD
rs7078530	RMVar_ID_878589	m6A	10	30697436	SVIL2P	exon	Prediction:(Low)	Functional Loss	G	A	0.3711	-0.0162	0.0019	1.10E-11	eBMD
rs10160116	RMVar_ID_839848	m6A	10	30849889	ZNF438	CDS	MeRIP-seq:(Medium)	Functional Loss	G	A	0.6019	0.0119	0.0019	4.20E-09	eBMD
rs10160116	RMVar_ID_701909	m6A	10	30849889	ZNF438	CDS	MeRIP-seq:(Medium)	Functional Loss	G	A	0.6019	0.0119	0.0019	4.20E-09	eBMD
rs912805	RMVar_ID_703728	m6A	10	43869258	LINC00840	exon	m6A-Label-seq:(High)	Functional Loss	T	C	0.5474	-0.0123	0.0018	5.90E-10	eBMD
rs912805	RMVar_ID_840197	m6A	10	43869258	LINC00840	exon	m6A-Label-seq:(High)	Functional Loss	T	C	0.5474	-0.0123	0.0018	5.90E-10	eBMD
rs11002730	RMVar_ID_213635	m6A	10	52611501	LOC10537830	exon	m6A-Label-seq:(High)	Functional Loss	A	C	0.4932	0.0309	0.0018	4.00E-53	eBMD
rs1838062	RMVar_ID_213636	m6A	10	52725689	lnc-DKK1-5	exon	m6A-Label-seq:(High)	Functional Loss	A	G	0.2437	-0.0170	0.0022	6.80E-13	eBMD
rs3824718	RMVar_ID_665596	m6A	10	79313756	ZMIZ1	3_UTR	MeRIP-seq:(Medium)	Functional Loss	C	G	0.6530	-0.0136	0.0019	5.80E-10	eBMD

rs3824718	RMVar_ID_841863	m6A	10	79313756	ZMIZ1	3_UTR	MeRIP-seq:(Medium)	Functional Loss	C	G	0.6530	-0.0136	0.0019	5.80E-10	eBMD
rs10887869	RMVar_ID_886280	m6A	10	80434933	PRXL2A	3_UTR	Prediction:(Low)	Functional Loss	A	G	0.4244	-0.0136	0.0019	3.60E-11	eBMD
rs10887869	RMVar_ID_886281	m6A	10	80434933	PRXL2A	3_UTR	Prediction:(Low)	Functional Loss	A	G	0.4244	-0.0136	0.0019	3.60E-11	eBMD
rs1042606	RMVar_ID_841957	m6A	10	110510918	DUSP5	3_UTR	MeRIP-seq:(Medium)	Functional Loss	A	C	0.3449	0.0144	0.0019	5.40E-12	eBMD
rs1042606	RMVar_ID_489721	m6A	10	110510918	DUSP5	3_UTR	MeRIP-seq:(Medium)	Functional Loss	A	C	0.3449	0.0144	0.0019	5.40E-12	eBMD
rs10749457	RMVar_ID_279731	m6A	10	122284546	BTBD16	intron	m6A-Label-seq:(High)	Functional Loss	A	T	0.5366	0.0139	0.0018	1.70E-09	eBMD
rs2421013	RMVar_ID_873632	m6A	10	122329491	BTBD16	CDS	Prediction:(Low)	Functional Loss	G	A	0.4925	0.0138	0.0018	7.70E-09	eBMD
rs3794153	RMVar_ID_860259	m6A	11	8730341	DENND2B	CDS	MeRIP-seq:(Medium)	Functional Loss	C	G	0.5287	-0.0164	0.0018	6.50E-15	eBMD
rs3794153	RMVar_ID_173554	m6A	11	8730341	ST5	CDS	MeRIP-seq:(Medium)	Functional Loss	C	G	0.5287	-0.0164	0.0018	6.50E-15	eBMD
rs2568044	RMVar_ID_116883	m1A	11	8980930	NRIP3	3_UTR	m1A-quant-seq:(High)	Functional Loss	T	C	0.2224	-0.0240	0.0022	2.40E-24	eBMD
rs2568044	RMVar_ID_58208	m1A	11	8980930	NRIP3	3_UTR	m1A-quant-seq:(High)	Functional Loss	T	C	0.2224	-0.0240	0.0022	2.40E-24	eBMD
rs273605	RMVar_ID_909338	m6A	11	30944310	DCDC1	intron	Prediction:(Low)	Functional Loss	A	T	0.3311	0.0126	0.0020	4.30E-09	eBMD
rs1051006	RMVar_ID_912539	m6A	11	47285038	MADD	CDS	Prediction:(Low)	Functional Gain	G	A	0.8245	-0.0211	0.0024	5.00E-15	eBMD
rs7104653	RMVar_ID_913374	m6A	11	50170422	AC109635.4	exon	Prediction:(Low)	Functional Loss	G	A	0.8219	-0.0190	0.0024	2.50E-14	eBMD
rs7104653	RMVar_ID_913375	m6A	11	50170422	AC109635.4	exon	Prediction:(Low)	Functional Loss	G	A	0.8219	-0.0190	0.0024	2.50E-14	eBMD
rs185498344	RMVar_ID_914514	m6A	11	58691535	AP000445.1	exon	Prediction:(Low)	Functional Loss	G	A	0.9865	-0.0591	0.0082	2.50E-11	eBMD
rs174537	RMVar_ID_915928	m6A	11	61785204	MYRF	intron	Prediction:(Low)	Functional Gain	G	T	0.6536	-0.0156	0.0019	2.00E-13	eBMD
rs4246215	RMVar_ID_859077	m6A	11	61796828	FEN1	3_UTR	miCLIP&DART-seq:(Hig	Functional Loss	G	T	0.6393	-0.0145	0.0019	2.80E-12	eBMD
rs4246215	RMVar_ID_158742	m6A	11	61796828	FEN1	3_UTR	miCLIP&DART-seq:(Hig	Functional Loss	G	T	0.6393	-0.0145	0.0019	2.80E-12	eBMD
rs4246215	RMVar_ID_158741	m6A	11	61796828	FEN1	3_UTR	miCLIP&DART-seq:(Hig	Functional Loss	G	T	0.6393	-0.0145	0.0019	2.80E-12	eBMD
rs3825071	RMVar_ID_918786	m6A	11	65444649	NEAT1	exon	Prediction:(Low)	Functional Gain	C	T	0.7825	-0.0190	0.0022	3.70E-12	eBMD
rs573673	RMVar_ID_861831	m6A	11	66640602	RBM4	intron	MeRIP-seq:(Medium)	Functional Loss	G	C	0.9518	-0.0317	0.0043	5.90E-12	eBMD
rs573673	RMVar_ID_164154	m6A	11	66640602	RBM4	intron	MeRIP-seq:(Medium)	Functional Loss	G	C	0.9518	-0.0317	0.0043	5.90E-12	eBMD
rs643892	RMVar_ID_57343	m1A	11	68380985	LRP5	intron	MeRIP-seq:(Medium)	Functional Loss	A	T	0.3486	-0.0250	0.0019	9.00E-31	eBMD
rs643892	RMVar_ID_57344	m1A	11	68380985	LRP5	intron	MeRIP-seq:(Medium)	Functional Loss	A	T	0.3486	-0.0250	0.0019	9.00E-31	eBMD
rs73516825	RMVar_ID_165630	m6A	11	68447539	LRP5	intron	MeRIP-seq:(Medium)	Functional Loss	C	A	0.8925	0.0243	0.0030	1.50E-14	eBMD
rs948852	RMVar_ID_165689	m6A	11	68683106	GAL	intron	MeRIP-seq:(Medium)	Functional Loss	G	T	0.3041	-0.0173	0.0020	1.80E-13	eBMD
rs948852	RMVar_ID_165688	m6A	11	68683106	GAL	intron	MeRIP-seq:(Medium)	Functional Loss	G	T	0.3041	-0.0173	0.0020	1.80E-13	eBMD
rs473997	RMVar_ID_921592	m6A	11	68872010	LINC02701	exon	Prediction:(Low)	Functional Gain	C	T	0.7160	-0.0133	0.0020	2.80E-10	eBMD
rs6590125	RMVar_ID_862315	m6A	11	125087287	SLC37A2	intron	m6A-Label-seq:(High)	Functional Loss	T	G	0.3513	0.0131	0.0019	2.40E-10	eBMD
rs6590125	RMVar_ID_475678	m6A	11	125087287	SLC37A2	intron	m6A-Label-seq:(High)	Functional Loss	T	G	0.3513	0.0131	0.0019	2.40E-10	eBMD
rs3017281	RMVar_ID_86580	m1A	11	125569527	EI24	5_UTR	MeRIP-seq:(Medium)	Functional Loss	T	C	0.1187	-0.0184	0.0028	3.30E-09	eBMD
rs3017281	RMVar_ID_116594	m1A	11	125569527	EI24	5_UTR	MeRIP-seq:(Medium)	Functional Loss	T	C	0.1187	-0.0184	0.0028	3.30E-09	eBMD
rs10773946	RMVar_ID_530516	m6A	12	1492555	ERC1	3_UTR	MeRIP-seq:(Medium)	Functional Loss	C	G	0.1604	-0.0202	0.0025	1.40E-11	eBMD
rs10773946	RMVar_ID_849060	m6A	12	1492555	ERC1	3_UTR	MeRIP-seq:(Medium)	Functional Loss	C	G	0.1604	-0.0202	0.0025	1.40E-11	eBMD

rs1841964	RMVar_ID_946661	m6A	12	28190074	LOC729291	exon	Prediction:(Low)	Functional Loss	G	A	0.7481	-0.0201	0.0021	7.10E-15	eBMD
rs4573721	RMVar_ID_546504	m6A	12	28194746	CCDC91	intron	m6A-Label-seq:(High)	Functional Loss	T	A	0.6850	0.0135	0.0020	1.90E-08	eBMD
rs4573721	RMVar_ID_850130	m6A	12	28194746	CCDC91	intron	m6A-Label-seq:(High)	Functional Loss	T	A	0.6850	0.0135	0.0020	1.90E-08	eBMD
rs4760610	RMVar_ID_950165	m6A	12	48024449	LOC10536975	intron	Prediction:(Low)	Functional Loss	C	T	0.7533	0.0173	0.0021	1.60E-10	eBMD
rs954	RMVar_ID_463746	m6A	12	48828436	CACNB3	3_UTR	MeRIP-seq:(Medium)	Functional Loss	T	C	0.7099	-0.0199	0.0020	5.80E-21	eBMD
rs7975791	RMVar_ID_950701	m6A	12	49019699	KMT2D	3_UTR	Prediction:(Low)	Functional Gain	C	T	0.9605	0.0385	0.0047	1.30E-11	eBMD
rs168220	RMVar_ID_507842	m6A	12	65920979	HMGA2	intron	m6A-Label-seq:(High)	Functional Loss	A	T	0.7661	-0.0135	0.0022	3.20E-08	eBMD
rs168220	RMVar_ID_507841	m6A	12	65920979	HMGA2	intron	m6A-Label-seq:(High)	Functional Loss	A	T	0.7661	-0.0135	0.0022	3.20E-08	eBMD
rs73138102	RMVar_ID_954142	m6A	12	77831168	NAV3	5_UTR	Prediction:(Low)	Functional Loss	A	T	0.6144	-0.0127	0.0019	7.10E-09	eBMD
rs3741768	RMVar_ID_939188	m6A	12	116008655	MED13L	CDS	Prediction:(Low)	Functional Loss	C	T	0.8244	0.0188	0.0024	1.30E-13	eBMD
rs2695481	RMVar_ID_54583	A-to-I	12	123158873	MPHOSPH9	intron	RNA-Seq:(High)	Functional Loss	T	C	0.2122	-0.0176	0.0023	5.00E-11	eBMD
rs2695481	RMVar_ID_35417	A-to-I	12	123158873	MPHOSPH9	intron	RNA-Seq:(High)	Functional Loss	T	C	0.2122	-0.0176	0.0023	5.00E-11	eBMD
rs2851438	RMVar_ID_54653	A-to-I	12	123159142	MPHOSPH9	intron	RNA-Seq:(High)	Functional Loss	T	C	0.2123	-0.0176	0.0023	4.50E-11	eBMD
rs2851438	RMVar_ID_35419	A-to-I	12	123159142	MPHOSPH9	intron	RNA-Seq:(High)	Functional Loss	T	C	0.2123	-0.0176	0.0023	4.50E-11	eBMD
rs2695479	RMVar_ID_35428	A-to-I	12	123204839	MPHOSPH9	intron	RNA-Seq:(High)	Functional Loss	T	C	0.2008	-0.0189	0.0023	4.40E-12	eBMD
rs11057192	RMVar_ID_35432	A-to-I	12	123208873	MPHOSPH9	intron	RNA-Seq:(High)	Functional Loss	T	C	0.2105	-0.0179	0.0023	2.50E-11	eBMD
rs11057192	RMVar_ID_54645	A-to-I	12	123208873	MPHOSPH9	intron	RNA-Seq:(High)	Functional Loss	T	C	0.2105	-0.0179	0.0023	2.50E-11	eBMD
rs11057192	RMVar_ID_35433	A-to-I	12	123208873	MPHOSPH9	intron	RNA-Seq:(High)	Functional Loss	T	C	0.2105	-0.0179	0.0023	2.50E-11	eBMD
rs1533703	RMVar_ID_544318	m6A	12	123257546	C12orf65	3_UTR	MeRIP-seq:(Medium)	Functional Loss	T	G	0.2102	-0.0184	0.0023	1.60E-11	eBMD
rs1533703	RMVar_ID_850444	m6A	12	123257546	MTRFR	3_UTR	MeRIP-seq:(Medium)	Functional Loss	T	G	0.2102	-0.0184	0.0023	1.60E-11	eBMD
rs1060105	RMVar_ID_852523	m6A	12	123321671	SBNO1	CDS	DART-seq:(High)	Functional Loss	C	T	0.7966	0.0192	0.0023	3.60E-12	eBMD
rs1060105	RMVar_ID_544369	m6A	12	123321671	SBNO1	CDS	DART-seq:(High)	Functional Loss	C	T	0.7966	0.0192	0.0023	3.60E-12	eBMD
rs411746	RMVar_ID_538120	m6A	13	42229881	DGKH	3_UTR	m6A-Label-seq:(High)	Functional Loss	T	C	0.0279	0.0398	0.0056	2.70E-09	eBMD
rs806293	RMVar_ID_692840	m6A	13	50238846	DLEU1	intron	m6A-Label-seq:(High)	Functional Loss	T	A	0.4674	-0.0128	0.0019	1.10E-09	eBMD
rs9557132	RMVar_ID_833994	m6A	13	99072023	DOCK9	intron	MeRIP-seq:(Medium)	Functional Loss	C	G	0.7990	-0.0283	0.0023	1.30E-28	eBMD
rs9557132	RMVar_ID_731210	m6A	13	99072023	DOCK9	intron	MeRIP-seq:(Medium)	Functional Loss	C	G	0.7990	-0.0283	0.0023	1.30E-28	eBMD
rs728982	RMVar_ID_731215	m6A	13	99087486	DOCK9-DT	exon	miCLIP:(High)	Functional Loss	T	G	0.7297	-0.0190	0.0021	3.10E-16	eBMD
rs7329444	RMVar_ID_731367	m6A	13	99969109	ZIC5	intron	miCLIP:(High)	Functional Loss	G	C	0.1961	0.0206	0.0023	7.20E-16	eBMD
rs3181248	RMVar_ID_974593	m6A	14	24338946	RIPK3	intron	Prediction:(Low)	Functional Loss	G	A	0.4125	-0.0133	0.0019	3.40E-09	eBMD
rs2275145	RMVar_ID_1597586	m7G	14	34773622	BAZ1A	CDS	MeRIP-seq:(Medium)	Functional Loss	G	A	0.4927	-0.0113	0.0019	2.30E-08	eBMD
rs2275145	RMVar_ID_1571302	m7G	14	34773622	BAZ1A	CDS	MeRIP-seq:(Medium)	Functional Loss	G	A	0.4927	-0.0113	0.0019	2.30E-08	eBMD
rs2275145	RMVar_ID_1571301	m7G	14	34773622	BAZ1A	CDS	MeRIP-seq:(Medium)	Functional Loss	G	A	0.4927	-0.0113	0.0019	2.30E-08	eBMD
rs10131094	RMVar_ID_25696	A-to-I	14	34819432	BAZ1A	intron	RNA-Seq:(High)	Functional Loss	T	C	0.3904	-0.0166	0.0019	7.60E-17	eBMD
rs2145031	RMVar_ID_975622	m6A	14	34874427	BAZ1A-AS1	exon	Prediction:(Low)	Functional Loss	A	C	0.7110	0.0163	0.0020	1.80E-13	eBMD
rs2273154	RMVar_ID_975647	m6A	14	34963988	SRP54-AS1	exon	Prediction:(Low)	Functional Gain	C	T	0.8104	-0.0176	0.0024	1.30E-10	eBMD

rs28384418	RMVar_ID_975656	m6A	14	34982888	SRP54	5_UTR	Prediction:(Low)	Functional Loss	C	A	0.7351	-0.0141	0.0021	1.50E-09	eBMD
rs7158047	RMVar_ID_798253	m6A	14	74922101	RPS6KL1	5_UTR	m6A-Seal-seq:(Medium)	Functional Loss	G	A	0.4123	-0.0123	0.0019	4.90E-10	eBMD
rs7158047	RMVar_ID_327376	m6A	14	74922101	RPS6KL1	5_UTR	m6A-Seal-seq:(Medium)	Functional Loss	G	A	0.4123	-0.0123	0.0019	4.90E-10	eBMD
rs12890371	RMVar_ID_983316	m6A	14	75127134	NEK9	5_UTR	Prediction:(Low)	Functional Gain	T	C	0.5246	-0.0141	0.0019	5.40E-11	eBMD
rs61217816	RMVar_ID_987115	m6A	14	95184708	CLMN	3_UTR	Prediction:(Low)	Functional Gain	T	C	0.9132	0.0251	0.0033	2.90E-12	eBMD
rs3814816	RMVar_ID_987118	m6A	14	95185430	CLMN	3_UTR	Prediction:(Low)	Functional Gain	G	A	0.9133	0.0252	0.0033	2.70E-12	eBMD
rs861530	RMVar_ID_989646	m6A	14	103707783	XRCC3	intron	Prediction:(Low)	Functional Loss	T	C	0.3003	0.0139	0.0020	4.80E-09	eBMD
rs4900592	RMVar_ID_989695	m6A	14	103710762	XRCC3	intron	Prediction:(Low)	Functional Loss	G	T	0.6733	-0.0154	0.0020	6.40E-12	eBMD
rs4900592	RMVar_ID_989694	m6A	14	103710762	XRCC3	intron	Prediction:(Low)	Functional Loss	G	T	0.6733	-0.0154	0.0020	6.40E-12	eBMD
rs2295146	RMVar_ID_206284	m6A	14	103733018	ZFYVE21	stop codon	DART-seq:(High)	Functional Loss	C	T	0.6045	-0.0119	0.0019	1.00E-08	eBMD
rs2295146	RMVar_ID_801447	m6A	14	103733018	ZFYVE21	stop codon	DART-seq:(High)	Functional Loss	C	T	0.6045	-0.0119	0.0019	1.00E-08	eBMD
rs732762	RMVar_ID_651150	m6A	15	51183689	MIR4713HG	exon	m6A-Label-seq:(High)	Functional Loss	T	C	0.3582	0.0216	0.0019	9.00E-25	eBMD
rs732762	RMVar_ID_651151	m6A	15	51183689	MIR4713HG	exon	m6A-Label-seq:(High)	Functional Loss	T	C	0.3582	0.0216	0.0019	9.00E-25	eBMD
rs12050772	RMVar_ID_651160	m6A	15	51252674	CYP19A1	intron	m6A-Label-seq:(High)	Functional Loss	T	G	0.5383	-0.0267	0.0019	7.40E-38	eBMD
rs8030077	RMVar_ID_1003424	m6A	15	63637037	HERC1	intron	Prediction:(Low)	Functional Gain	T	A	0.8752	-0.0223	0.0028	1.00E-13	eBMD
rs8031179	RMVar_ID_96606	m1A	15	65659696	DENND4A	CDS	m1A-quant-seq:(High)	Functional Loss	T	C	0.5715	0.0126	0.0019	1.60E-09	eBMD
rs8031179	RMVar_ID_96607	m1A	15	65659696	DENND4A	CDS	m1A-quant-seq:(High)	Functional Loss	T	C	0.5715	0.0126	0.0019	1.60E-09	eBMD
rs2727100	RMVar_ID_1004240	m6A	15	65771221	DENND4A	intron	Prediction:(Low)	Functional Loss	G	A	0.8295	0.0224	0.0025	3.30E-15	eBMD
rs3751655	RMVar_ID_567123	m6A	15	90228518	SEMA4B	CDS	MeRIP-seq:(Medium)	Functional Loss	T	G	0.3480	0.0123	0.0020	4.40E-08	eBMD
rs3751655	RMVar_ID_774378	m6A	15	90228518	SEMA4B	CDS	MeRIP-seq:(Medium)	Functional Loss	T	G	0.3480	0.0123	0.0020	4.40E-08	eBMD
rs3751655	RMVar_ID_567124	m6A	15	90228518	SEMA4B	CDS	MeRIP-seq:(Medium)	Functional Loss	T	G	0.3480	0.0123	0.0020	4.40E-08	eBMD
rs7174198	RMVar_ID_1013412	m6A	15	90539952	CRTC3	intron	Prediction:(Low)	Functional Gain	C	T	0.7928	0.0174	0.0023	2.10E-11	eBMD
rs112865841	RMVar_ID_1016623	m6A	16	384348	LOC10537103	exon	Prediction:(Low)	Functional Loss	G	T	0.7932	0.0174	0.0023	2.60E-11	eBMD
rs7203729	RMVar_ID_72951	m1A	16	2090009	PKD1	CDS	MeRIP-seq:(Medium)	Functional Loss	A	G	0.8152	-0.0201	0.0024	4.10E-15	eBMD
rs7203729	RMVar_ID_72950	m1A	16	2090009	PKD1	CDS	MeRIP-seq:(Medium)	Functional Loss	A	G	0.8152	-0.0201	0.0024	4.10E-15	eBMD
rs7203729	RMVar_ID_72952	m1A	16	2090009	PKD1	CDS	MeRIP-seq:(Medium)	Functional Loss	A	G	0.8152	-0.0201	0.0024	4.10E-15	eBMD
rs2531988	RMVar_ID_335564	m6A	16	3978416	ADCY9	intron	m6A-Label-seq:(High)	Functional Loss	T	G	0.1656	-0.0232	0.0025	1.80E-16	eBMD
rs2531988	RMVar_ID_335563	m6A	16	3978416	ADCY9	intron	m6A-Label-seq:(High)	Functional Loss	T	G	0.1656	-0.0232	0.0025	1.80E-16	eBMD
rs2283479	RMVar_ID_1021977	m6A	16	4264526	TFAP4	intron	Prediction:(Low)	Functional Loss	C	A	0.4980	0.0115	0.0019	1.40E-09	eBMD
rs2283479	RMVar_ID_1021976	m6A	16	4264526	TFAP4	intron	Prediction:(Low)	Functional Loss	C	A	0.4980	0.0115	0.0019	1.40E-09	eBMD
rs3803716	RMVar_ID_264706	m6A	16	24791003	TNRC6A	CDS	miCLIP:(High)	Functional Loss	C	T	0.8044	0.0141	0.0023	2.30E-08	eBMD
rs3803716	RMVar_ID_787406	m6A	16	24791003	TNRC6A	CDS	miCLIP:(High)	Functional Loss	C	T	0.8044	0.0141	0.0023	2.30E-08	eBMD
rs7201	RMVar_ID_113153	m1A	16	55505702	MMP2	3_UTR	m1A-quant-seq:(High)	Functional Loss	A	C	0.5411	-0.0114	0.0019	1.30E-09	eBMD
rs7201	RMVar_ID_95218	m1A	16	55505702	MMP2	3_UTR	m1A-quant-seq:(High)	Functional Loss	A	C	0.5411	-0.0114	0.0019	1.30E-09	eBMD
rs61733768	RMVar_ID_1037335	m6A	16	67180056	KIAA0895L	CDS	Prediction:(Low)	Functional Loss	G	A	0.9708	0.0760	0.0055	7.70E-36	eBMD

rs8053031	RMVar_ID_547385	m6A	16	67264260	SLC9A5	intron	m6A-Label-seq:(High)	Functional Loss	T	G	0.9690	0.0696	0.0053	2.70E-34	eBMD
rs143837268	RMVar_ID_1037747	m6A	16	67620779	CTCF	CDS	Prediction:(Low)	Functional Gain	C	T	0.9818	0.0561	0.0069	1.00E-15	eBMD
rs118016432	RMVar_ID_35774	A-to-I	16	67772793	RANBP10	intron	RNA-Seq:(High)	Functional Loss	T	C	0.9696	0.0321	0.0054	1.90E-09	eBMD
rs216196	RMVar_ID_673575	m6A	17	2299651	SMG6	CDS	MeRIP-seq:(Medium)	Functional Loss	T	C	0.7229	0.0457	0.0021	1.20E-88	eBMD
rs216196	RMVar_ID_778992	m6A	17	2299651	SMG6	CDS	MeRIP-seq:(Medium)	Functional Loss	T	C	0.7229	0.0457	0.0021	1.20E-88	eBMD
rs1885987	RMVar_ID_673582	m6A	17	2299731	SMG6	CDS	MeRIP-seq:(Medium)	Functional Loss	T	G	0.6292	-0.0138	0.0019	7.70E-10	eBMD
rs1885987	RMVar_ID_673583	m6A	17	2299731	SMG6	CDS	MeRIP-seq:(Medium)	Functional Loss	T	G	0.6292	-0.0138	0.0019	7.70E-10	eBMD
rs1885987	RMVar_ID_782642	m6A	17	2299731	SMG6	CDS	MeRIP-seq:(Medium)	Functional Loss	T	G	0.6292	-0.0138	0.0019	7.70E-10	eBMD
rs216194	RMVar_ID_45835	A-to-I	17	2300054	SMG6	CDS	RNA-Seq:(High)	Functional Loss	T	C	0.1544	0.0190	0.0026	1.10E-11	eBMD
rs216194	RMVar_ID_53094	A-to-I	17	2300054	SMG6	CDS	RNA-Seq:(High)	Functional Loss	T	C	0.1544	0.0190	0.0026	1.10E-11	eBMD
rs2248821	RMVar_ID_1048429	m6A	17	2365021	SGSM2	CDS	Prediction:(Low)	Functional Gain	G	A	0.5414	0.0128	0.0019	7.80E-10	eBMD
rs4450459	RMVar_ID_46132	A-to-I	17	7493298	POLR2A	intron	RNA-Seq:(High)	Functional Loss	A	G	0.0379	0.0293	0.0048	1.80E-08	eBMD
rs4450459	RMVar_ID_46131	A-to-I	17	7493298	POLR2A	intron	RNA-Seq:(High)	Functional Loss	A	G	0.0379	0.0293	0.0048	1.80E-08	eBMD
rs4791806	RMVar_ID_677414	m6A	17	7825866	DNAH2	intron	m6A-Label-seq:(High)	Functional Loss	A	G	0.1848	-0.0185	0.0024	1.70E-12	eBMD
rs62623385	RMVar_ID_677678	m6A	17	7944520	CNTROB	CDS	miCLIP:(High)	Functional Loss	A	T	0.9658	-0.0346	0.0051	1.90E-08	eBMD
rs62066870	RMVar_ID_198668	m6A	17	20160445	SPECC1	intron	m6A-Label-seq:(High)	Functional Loss	A	C	0.3879	-0.0107	0.0019	4.20E-08	eBMD
rs62066870	RMVar_ID_784802	m6A	17	20160445	SPECC1	intron	m6A-Label-seq:(High)	Functional Loss	A	C	0.3879	-0.0107	0.0019	4.20E-08	eBMD
rs4564638	RMVar_ID_1060931	m6A	17	31526528	RAB11FIP4	intron	Prediction:(Low)	Functional Gain	T	C	0.3606	0.0118	0.0019	4.40E-08	eBMD
rs668799	RMVar_ID_1066384	m6A	17	42564192	COASY	intron	Prediction:(Low)	Functional Loss	C	T	0.7207	-0.0131	0.0021	1.50E-09	eBMD
rs1983490	RMVar_ID_1067271	m6A	17	43705591	LINC02594	exon	Prediction:(Low)	Functional Loss	G	A	0.6200	-0.0385	0.0019	1.40E-68	eBMD
rs1983490	RMVar_ID_1067271	m6A	17	43705591	LINC02594	exon	Prediction:(Low)	Functional Loss	G	A	0.6200	0.0499	0.0068	3.60E-13	Fracture
rs2342312	RMVar_ID_729724	m6A	17	43719885	WHSC1L2P	exon	MeRIP-seq:(Medium)	Functional Loss	C	G	0.6813	-0.0408	0.0020	4.70E-72	eBMD
rs2342312	RMVar_ID_781655	m6A	17	43719885	WHSC1L2P	exon	MeRIP-seq:(Medium)	Functional Loss	C	G	0.6813	-0.0408	0.0020	4.70E-72	eBMD
rs2342312	RMVar_ID_729725	m6A	17	43719885	WHSC1L2P	exon	MeRIP-seq:(Medium)	Functional Loss	C	G	0.6813	-0.0408	0.0020	4.70E-72	eBMD
rs2342312	RMVar_ID_729725	m6A	17	43719885	WHSC1L2P	exon	MeRIP-seq:(Medium)	Functional Loss	C	G	0.6812	0.0518	0.0071	4.40E-13	Fracture
rs2342312	RMVar_ID_729724	m6A	17	43719885	WHSC1L2P	exon	MeRIP-seq:(Medium)	Functional Loss	C	G	0.6812	0.0518	0.0071	4.40E-13	Fracture
rs2342312	RMVar_ID_781655	m6A	17	43719885	WHSC1L2P	exon	MeRIP-seq:(Medium)	Functional Loss	C	G	0.6812	0.0518	0.0071	4.40E-13	Fracture
rs2537	RMVar_ID_1592717	m7G	17	43875439	MPP2	3_UTR	MeRIP-seq:(Medium)	Functional Loss	G	A	0.9417	0.0396	0.0040	5.20E-18	eBMD
rs228758	RMVar_ID_729828	m6A	17	44070835	G6PC3	5_UTR	MeRIP-seq:(Medium)	Functional Loss	C	T	0.4528	0.0213	0.0019	5.50E-24	eBMD
rs227584	RMVar_ID_112460	m1A	17	44148179	HROB	CDS	m1A-quant-seq:(High)	Functional Loss	A	C	0.7078	-0.0318	0.0020	1.70E-41	eBMD
rs227584	RMVar_ID_109095	m1A	17	44148179	HROB	CDS	m1A-quant-seq:(High)	Functional Loss	A	C	0.7078	-0.0318	0.0020	1.70E-41	eBMD
rs227584	RMVar_ID_109095	m1A	17	44148179	HROB	CDS	m1A-quant-seq:(High)	Functional Loss	A	C	0.7078	0.0466	0.0072	6.90E-11	Fracture
rs227584	RMVar_ID_112460	m1A	17	44148179	HROB	CDS	m1A-quant-seq:(High)	Functional Loss	A	C	0.7078	0.0466	0.0072	6.90E-11	Fracture
rs8079310	RMVar_ID_1067620	m6A	17	44192930	ATXN7L3	3_UTR	Prediction:(Low)	Functional Gain	A	G	0.7068	-0.0322	0.0020	1.80E-42	eBMD
rs8079310	RMVar_ID_1067620	m6A	17	44192930	ATXN7L3	3_UTR	Prediction:(Low)	Functional Gain	A	G	0.7068	0.0461	0.0072	1.20E-10	Fracture

rs2526011	RMVar_ID_109112	m1A	17	44210760	UBTF	intron	MeRIP-seq:(Medium)	Functional Loss	T	G	0.9057	-0.0296	0.0032	1.10E-15	eBMD
rs2526011	RMVar_ID_109113	m1A	17	44210760	UBTF	intron	MeRIP-seq:(Medium)	Functional Loss	T	G	0.9057	-0.0296	0.0032	1.10E-15	eBMD
rs2526011	RMVar_ID_109111	m1A	17	44210760	UBTF	intron	MeRIP-seq:(Medium)	Functional Loss	T	G	0.9057	-0.0296	0.0032	1.10E-15	eBMD
rs2526011	RMVar_ID_112590	m1A	17	44210760	UBTF	intron	MeRIP-seq:(Medium)	Functional Loss	T	G	0.9057	-0.0296	0.0032	1.10E-15	eBMD
rs2239918	RMVar_ID_591762	m6A	17	45091741	NMT1	intron	m6A-Label-seq:(High)	Functional Loss	T	A	0.5464	0.0112	0.0019	4.70E-08	eBMD
rs1132898	RMVar_ID_1068236	m6A	17	45093784	NMT1	CDS	Prediction:(Low)	Functional Gain	C	T	0.7855	0.0208	0.0023	2.20E-15	eBMD
rs1132898	RMVar_ID_1068237	m6A	17	45093784	NMT1	CDS	Prediction:(Low)	Functional Gain	C	T	0.7855	0.0208	0.0023	2.20E-15	eBMD
rs1879581	RMVar_ID_592128	m6A	17	45468529	PLEKHM1	CDS	MeRIP-seq:(Medium)	Functional Loss	T	C	0.8156	0.0242	0.0024	1.50E-20	eBMD
rs385691	RMVar_ID_1068835	m6A	17	45648759	CRHR1	intron	Prediction:(Low)	Functional Loss	A	C	0.7751	0.0232	0.0022	2.60E-21	eBMD
rs385691	RMVar_ID_1068836	m6A	17	45648759	CRHR1	intron	Prediction:(Low)	Functional Loss	A	C	0.7751	0.0232	0.0022	2.60E-21	eBMD
rs366858	RMVar_ID_1068841	m6A	17	45649244	CRHR1	intron	Prediction:(Low)	Functional Loss	C	T	0.7751	0.0232	0.0022	2.70E-21	eBMD
rs76324150	RMVar_ID_1068953	m6A	17	45895887	MAPT	intron	Prediction:(Low)	Functional Loss	C	T	0.7749	0.0231	0.0022	5.00E-21	eBMD
rs4792891	RMVar_ID_1068959	m6A	17	45896140	MAPT	intron	Prediction:(Low)	Functional Gain	T	G	0.6622	0.0165	0.0020	7.30E-16	eBMD
rs17650901	RMVar_ID_592266	m6A	17	45962325	MAPT	5_UTR	MeRIP-seq:(Medium)	Functional Loss	A	G	0.7749	0.0231	0.0022	7.10E-21	eBMD
rs7350928	RMVar_ID_1583196	m7G	17	46030734	KANSL1	3_UTR	MeRIP-seq:(Medium)	Functional Loss	C	T	0.7749	0.0230	0.0022	1.00E-20	eBMD
rs7350928	RMVar_ID_1583197	m7G	17	46030734	KANSL1	3_UTR	MeRIP-seq:(Medium)	Functional Loss	C	T	0.7749	0.0230	0.0022	1.00E-20	eBMD
rs17574425	RMVar_ID_1069086	m6A	17	46031828	KANSL1	intron	Prediction:(Low)	Functional Gain	C	G	0.7749	0.0231	0.0022	9.30E-21	eBMD
rs1881193	RMVar_ID_39617	A-to-I	17	46171403	KANSL1	CDS	RNA-Seq:(High)	Functional Loss	T	C	0.7744	0.0232	0.0022	4.80E-21	eBMD
rs1881193	RMVar_ID_39616	A-to-I	17	46171403	KANSL1	CDS	RNA-Seq:(High)	Functional Loss	T	C	0.7744	0.0232	0.0022	4.80E-21	eBMD
rs58872582	RMVar_ID_609051	m6A	17	60810499	BCAS3	intron	MeRIP-seq:(Medium)	Functional Loss	A	G	0.8965	-0.0519	0.0030	1.50E-48	eBMD
rs1991401	RMVar_ID_1075834	m6A	17	64506321	DDX5	5_UTR	Prediction:(Low)	Functional Loss	A	G	0.6861	0.0171	0.0020	2.40E-17	eBMD
rs34587622	RMVar_ID_629638	m6A	17	77402414	SEPTIN9	CDS	MeRIP-seq:(Medium)	Functional Loss	C	T	0.8879	0.0173	0.0029	1.90E-08	eBMD
rs8074498	RMVar_ID_633489	m6A	17	81996666	ASPSCR1	CDS	miCLIP:(High)	Functional Loss	T	A	0.4183	0.0114	0.0019	8.00E-10	eBMD
rs12453001	RMVar_ID_1084606	m6A	17	82577259	FOXK2	intron	Prediction:(Low)	Functional Loss	T	A	0.3648	-0.0186	0.0019	4.80E-20	eBMD
rs61735998	RMVar_ID_1090719	m6A	18	36709302	FHOD3	CDS	Prediction:(Low)	Functional Loss	G	T	0.9743	-0.0412	0.0059	3.30E-09	eBMD
rs978572	RMVar_ID_566098	m6A	18	62254957	RELCH	intron	m6A-Label-seq:(High)	Functional Loss	T	C	0.2663	-0.0141	0.0021	3.00E-10	eBMD
rs978572	RMVar_ID_566097	m6A	18	62254957	RELCH	intron	m6A-Label-seq:(High)	Functional Loss	T	C	0.2663	-0.0141	0.0021	3.00E-10	eBMD
rs3180287	RMVar_ID_1608266	m7G	19	804396	PTBP1	CDS	MeRIP-seq:(Medium)	Functional Loss	C	T	0.8616	0.0225	0.0027	1.50E-15	eBMD
rs3180287	RMVar_ID_1600467	m7G	19	804396	PTBP1	CDS	MeRIP-seq:(Medium)	Functional Loss	C	T	0.8616	0.0225	0.0027	1.50E-15	eBMD
rs3180287	RMVar_ID_1569293	m7G	19	804396	PTBP1	CDS	MeRIP-seq:(Medium)	Functional Loss	C	T	0.8616	0.0225	0.0027	1.50E-15	eBMD
rs3180287	RMVar_ID_1569292	m7G	19	804396	PTBP1	CDS	MeRIP-seq:(Medium)	Functional Loss	C	T	0.8616	0.0225	0.0027	1.50E-15	eBMD
rs4807205	RMVar_ID_417454	m6A	19	2167879	DOT1L	intron	m6A-Label-seq:(High)	Functional Loss	A	G	0.5389	0.0148	0.0019	8.00E-13	eBMD
rs10405636	RMVar_ID_55387	m1A	19	18427932	SSBP4	CDS	MeRIP-seq:(Medium)	Functional Loss	A	C	0.6439	-0.0139	0.0019	1.10E-12	eBMD
rs10405636	RMVar_ID_111580	m1A	19	18427932	SSBP4	CDS	MeRIP-seq:(Medium)	Functional Loss	A	C	0.6439	-0.0139	0.0019	1.10E-12	eBMD
rs2041109	RMVar_ID_309220	m6A	19	57291437	ZNF460	CDS	MeRIP-seq:(Medium)	Functional Loss	C	T	0.7392	0.0113	0.0021	9.00E-09	eBMD

rs2041109	RMVar_ID_768500	m6A	19	57291437	ZNF460	CDS	MeRIP-seq:(Medium)	Functional Loss	C	T	0.7392	0.0113	0.0021	9.00E-09	eBMD
rs2041109	RMVar_ID_309221	m6A	19	57291437	ZNF460	CDS	MeRIP-seq:(Medium)	Functional Loss	C	T	0.7392	0.0113	0.0021	9.00E-09	eBMD
rs235768	RMVar_ID_856230	m6A	20	6778468	BMP2	CDS	m6A-Label-seq&miCLIP:	Functional Loss	A	T	0.3904	-0.0126	0.0019	2.10E-09	eBMD
rs235768	RMVar_ID_395129	m6A	20	6778468	BMP2	CDS	m6A-Label-seq&miCLIP:	Functional Loss	A	T	0.3904	-0.0126	0.0019	2.10E-09	eBMD
rs235768	RMVar_ID_395128	m6A	20	6778468	BMP2	CDS	m6A-Label-seq&miCLIP:	Functional Loss	A	T	0.3904	-0.0126	0.0019	2.10E-09	eBMD
rs13037675	RMVar_ID_395152	m6A	20	6779058	BMP2	CDS	miCLIP:(High)	Functional Loss	C	T	0.9274	-0.0402	0.0036	1.20E-20	eBMD
rs6040041	RMVar_ID_395358	m6A	20	10603861	SLX4IP	intron	m6A-Label-seq:(High)	Functional Loss	A	G	0.3268	0.0120	0.0020	3.10E-08	eBMD
rs6040041	RMVar_ID_395359	m6A	20	10603861	SLX4IP	intron	m6A-Label-seq:(High)	Functional Loss	A	G	0.3268	0.0120	0.0020	3.10E-08	eBMD
rs6040041	RMVar_ID_855124	m6A	20	10603861	SLX4IP	intron	m6A-Label-seq:(High)	Functional Loss	A	G	0.3268	0.0120	0.0020	3.10E-08	eBMD
rs61364340	RMVar_ID_395410	m6A	20	10623983	SLX4IP	3_UTR	miCLIP:(High)	Functional Loss	A	G	0.9190	0.0356	0.0034	2.90E-22	eBMD
rs80233229	RMVar_ID_395455	m6A	20	10666195	JAG1	intron	m6A-Label-seq:(High)	Functional Loss	T	C	0.8894	0.0430	0.0030	4.70E-38	eBMD
rs7353058	RMVar_ID_395464	m6A	20	10794845	LOC10798539	exon	m6A-Label-seq:(High)	Functional Loss	T	C	0.6370	0.0134	0.0019	2.20E-09	eBMD
rs7353058	RMVar_ID_856310	m6A	20	10794845	LOC10798539	exon	m6A-Label-seq:(High)	Functional Loss	T	C	0.6370	0.0134	0.0019	2.20E-09	eBMD
rs291700	RMVar_ID_107996	m1A	20	33394043	CDK5RAP1	CDS	m1A-quant-seq:(High)	Functional Loss	T	C	0.3179	0.0149	0.0020	6.80E-15	eBMD
rs291700	RMVar_ID_116480	m1A	20	33394043	CDK5RAP1	CDS	m1A-quant-seq:(High)	Functional Loss	T	C	0.3179	0.0149	0.0020	6.80E-15	eBMD
rs2295354	RMVar_ID_719358	m6A	20	34768738	NCOA6	intron	m6A-Label-seq:(High)	Functional Loss	T	C	0.6140	-0.0196	0.0019	2.10E-20	eBMD
rs2295354	RMVar_ID_857291	m6A	20	34768738	NCOA6	intron	m6A-Label-seq:(High)	Functional Loss	T	C	0.6140	-0.0196	0.0019	2.10E-20	eBMD
rs8501	RMVar_ID_108080	m1A	20	35002781	TRPC4AP	3_UTR	m1A-quant-seq:(High)	Functional Loss	T	C	0.8223	-0.0253	0.0024	2.70E-22	eBMD
rs8501	RMVar_ID_116498	m1A	20	35002781	TRPC4AP	3_UTR	m1A-quant-seq:(High)	Functional Loss	T	C	0.8223	-0.0253	0.0024	2.70E-22	eBMD
rs8501	RMVar_ID_108079	m1A	20	35002781	TRPC4AP	3_UTR	m1A-quant-seq:(High)	Functional Loss	T	C	0.8223	-0.0253	0.0024	2.70E-22	eBMD
rs6072351	RMVar_ID_273226	m6A	20	41359500	LPIN3	3_UTR	MeRIP-seq:(Medium)	Functional Loss	G	C	0.7757	0.0201	0.0022	2.90E-16	eBMD
rs995533	RMVar_ID_1216584	m6A	21	27367625	EIF4A1P1	exon	Prediction:(Low)	Functional Gain	C	G	0.3786	-0.0137	0.0019	4.20E-11	eBMD
rs11556025	RMVar_ID_89917	m1A	22	28772767	CCDC117	5_UTR	MeRIP-seq:(Medium)	Functional Loss	T	C	0.7038	0.0181	0.0020	2.10E-15	eBMD
rs11556025	RMVar_ID_89916	m1A	22	28772767	CCDC117	5_UTR	MeRIP-seq:(Medium)	Functional Loss	T	C	0.7038	0.0181	0.0020	2.10E-15	eBMD
rs11556025	RMVar_ID_112105	m1A	22	28772767	CCDC117	5_UTR	MeRIP-seq:(Medium)	Functional Loss	T	C	0.7038	0.0181	0.0020	2.10E-15	eBMD
rs6005881	RMVar_ID_516495	m6A	22	28787145	CCDC117	3_UTR	DART-seq:(High)	Functional Loss	A	G	0.7017	0.0179	0.0020	1.80E-15	eBMD
rs6005881	RMVar_ID_776539	m6A	22	28787145	CCDC117	3_UTR	DART-seq:(High)	Functional Loss	A	G	0.7017	0.0179	0.0020	1.80E-15	eBMD
rs2072797	RMVar_ID_1608854	m7G	22	38738202	SUN2	CDS	MeRIP-seq:(Medium)	Functional Loss	C	T	0.8859	0.0280	0.0029	1.10E-15	eBMD
rs2072797	RMVar_ID_1577515	m7G	22	38738202	SUN2	CDS	MeRIP-seq:(Medium)	Functional Loss	C	T	0.8859	0.0280	0.0029	1.10E-15	eBMD
rs2072797	RMVar_ID_1602810	m7G	22	38738202	SUN2	CDS	MeRIP-seq:(Medium)	Functional Loss	C	T	0.8859	0.0280	0.0029	1.10E-15	eBMD
rs6007010	RMVar_ID_1228193	m6A	22	45387026	SMC1B	CDS	Prediction:(Low)	Functional Gain	T	C	0.6534	0.0121	0.0020	1.90E-11	eBMD
rs2142661	RMVar_ID_1228227	m6A	22	45426000	RIBC2	CDS	Prediction:(Low)	Functional Gain	C	T	0.8369	0.0160	0.0025	7.20E-10	eBMD
rs1535189	RMVar_ID_185028	m6A	22	49855483	ZBED4	intron	m6A-Label-seq:(High)	Functional Loss	T	C	0.1277	-0.0209	0.0028	4.20E-14	eBMD
rs910797	RMVar_ID_59290	m1A	22	49884235	ZBED4	CDS	m1A-quant-seq:(High)	Functional Loss	A	G	0.1043	-0.0229	0.0030	1.80E-13	eBMD
rs910797	RMVar_ID_112255	m1A	22	49884235	ZBED4	CDS	m1A-quant-seq:(High)	Functional Loss	A	G	0.1043	-0.0229	0.0030	1.80E-13	eBMD

rs11545763	RMVar_ID_52958	A-to-I	22	49925522	CRELD2	CDS	RNA-Seq:(High)	Functional Loss	A	G	0.8930	0.0219	0.0030	7.50E-13	eBMD
rs11545763	RMVar_ID_5550	A-to-I	22	49925522	CRELD2	CDS	RNA-Seq:(High)	Functional Loss	A	G	0.8930	0.0219	0.0030	7.50E-13	eBMD
rs28379706	RMVar_ID_777655	m6A	22	50289635	PLXNB2	CDS	miCLIP:(High)	Functional Loss	T	C	0.6052	0.0136	0.0019	2.80E-11	eBMD
rs28379706	RMVar_ID_185842	m6A	22	50289635	PLXNB2	CDS	miCLIP:(High)	Functional Loss	T	C	0.6052	0.0136	0.0019	2.80E-11	eBMD
rs28455041	RMVar_ID_59387	m1A	22	50293474	PLXNB2	intron	MeRIP-seq:(Medium)	Functional Loss	T	C	0.6857	0.0135	0.0020	9.60E-11	eBMD
rs1997715	RMVar_ID_1479876	m6A	X	57596176	ZXDB	3_UTR	Prediction:(Low)	Functional Loss	G	A	0.7767	-0.0225	0.0023	1.57E-23	eBMD

Supplementary Table S2 RNAm_SNPs affected expression of key OP susceptibility genes

						Affected key OP genes		
SNP	CHR	Position	eqtl	Local gene	If cis effect	OP gene	P value	Tissue
rs2229503	2	54631527	GTEx2015_v6	SPTBN1	1	SPTBN1	1.80E-07	Artery_Aorta
							6.30E-09	Artery_Tibial
							4.90E-06	Esophagus_Mucosa
							1.42E-07	Esophagus_Muscularis
							3.10E-05	Whole_Blood
rs75072999	4	850993	GTEx2015_v6	GAK	1	FGFRL1	7.97E-08	Thyroid
rs6815946	4	1001517	GTEx2015_v6	IDUA	0	FGFRL1	9.76E-12	Lymphoblastoid
rs115790973	4	1002772	GTEx2015_v6	IDUA	0	FGFRL1	6.50E-11	Lymphoblastoid
rs3796619	4	1101493	GTEx2015_v6	RNF212	1	FGFRL1	3.28E-07	Cells_Transformed_fibroblasts
rs643892	11	68380985	GTEx2015_v6	LRP5	1	LRP5	1.04E-06	Skin_Sun_Exposed_Lower_leg
rs1983490	17	43705589	GTEx2015_v6	LINC02594	0	SOST	3.15E-06	Artery_Tibial
							5.96E-06	Brain_Cortex
							4.03E-06	Brain_Frontal_Cortex_BA9
rs227584	17	44148179	GTEx2015_v6	HROB	0	HDAC5	9.21E-07	Esophagus_Mucosa
rs8079310	17	44192923	GTEx2015_v6	ATXN7L3	0	HDAC5	7.05E-07	Esophagus_Mucosa
rs76324150	17	45895867	GTEx2015_v6	MAPT	1	WNT3	2.33E-07	Adrenal_Gland
							5.41E-09	Artery_Aorta
							6.15E-06	Artery_Coronary
							6.84E-06	Artery_Tibial
							1.83E-06	Esophagus_Muscularis
							1.52E-06	Heart_Atrial_Appendage
							6.60E-07	Lung
							8.36E-07	Nerve_Tibial
							5.17E-10	Pancreas
							1.74E-05	Testis
							2.35E-15	Thyroid
rs4792891	17	45896132	GTEx2015_v6	MAPT	1	WNT3	1.58E-06	Artery_Aorta
							4.59E-06	Heart_Atrial_Appendage
							6.48E-06	Pancreas
							1.37E-10	Thyroid
rs17650901	17	45962325	GTEx2015_v6	MAPT	1	WNT3	2.33E-07	Adrenal_Gland
							5.41E-09	Artery_Aorta
							6.15E-06	Artery_Coronary
							6.84E-06	Artery_Tibial
							1.83E-06	Esophagus_Muscularis
							1.52E-06	Heart_Atrial_Appendage
							6.60E-07	Lung
							8.36E-07	Nerve_Tibial
							5.17E-10	Pancreas
							1.74E-05	Testis
							2.35E-15	Thyroid
rs7350928	17	46030734	GTEx2015_v6	KANSL1	1	WNT3	2.33E-07	Adrenal_Gland
							5.42E-09	Artery_Aorta
							6.15E-06	Artery_Coronary
							6.84E-06	Artery_Tibial
							1.83E-06	Esophagus_Muscularis
							1.52E-06	Heart_Atrial_Appendage
							6.60E-07	Lung

						8.36E-07	Nerve_Tibial
						5.16E-10	Pancreas
						1.74E-05	Testis
						2.34E-15	Thyroid
rs17574425	17	46031822	GTEx2015_v6	KANSL1	1	WNT3	2.33E-07	Adrenal_Gland
						5.41E-09	Artery_Aorta
						6.15E-06	Artery_Coronary
						6.84E-06	Artery_Tibial
						1.83E-06	Esophagus_Muscularis
						1.52E-06	Heart_Atrial_Appendage
						6.60E-07	Lung
						8.36E-07	Nerve_Tibial
						5.17E-10	Pancreas
						1.74E-05	Testis
						2.35E-15	Thyroid
rs978572	18	62254957	GTEx2015_v6	RELCH	0	TNFRSF11A	5.44E-09	Esophagus_Mucosa
						8.75E-06	Pancreas
						6.17E-12	Skin_Sun_Exposed_Lower_leg

Supplementary Table S3 Gene expression associated with BMD in SMR analysis

Gene	probeID	ProbeChr	Probe_bp	b_SMR	se_SMR	p_SMR	p_HEIDI	Tissue
MTOR	ILMN_1769031	1	11166911	-0.0884	0.0169	1.62E-07	4.33E-02	Whole blood CAGE
MTOR	ENSG00000198793.8	1	11244578	-0.0685	0.0114	1.96E-09	2.57E-02	Whole Blood GTEx
CDC42	ILMN_1675156	1	22379209	-0.0389	0.0030	1.48E-37	7.42E-25	Whole blood westra
SCMH1	ILMN_1714738	1	41651847	0.1411	0.0305	3.79E-06	2.36E-01	Whole blood westra
ST7L	ILMN_1659926	1	113084506	0.0765	0.0122	3.26E-10	2.89E-08	Whole blood westra
ST7L	ILMN_1659926	1	113084507	0.0480	0.0073	6.36E-11	4.05E-09	Whole blood CAGE
ST7L	ILMN_2410113	1	113098585	0.1021	0.0187	4.80E-08	1.58E-05	Whole blood westra
ST7L	ILMN_2410113	1	113098586	0.0603	0.0103	4.08E-09	5.63E-11	Whole blood CAGE
ST7L	ENSG00000007341.14	1	113122614	0.0231	0.0035	6.35E-11	1.37E-07	Adipose Visceral Omentum
ST7L	ENSG00000007341.14	1	113122614	0.0219	0.0032	4.73E-12	1.09E-15	Adipose Subcutaneous
ST7L	ENSG00000007341.14	1	113122614	0.0408	0.0069	3.17E-09	1.65E-11	Muscle Skeletal
ST7L	ENSG00000007341.14	1	113122614	0.0462	0.0091	4.47E-07	1.89E-01	Whole Blood GTEx
PSEN2	ILMN_1714417	1	227083641	0.0684	0.0147	3.11E-06	5.92E-02	Whole blood westra
JMJD4	ILMN_1692896	1	227919163	-0.0249	0.0053	2.76E-06	8.64E-09	Whole blood westra
TRMT61B	ILMN_1706558	2	29073207	0.0677	0.0119	1.43E-08	1.69E-03	Whole blood CAGE
TRMT61B	ENSG00000171103.6	2	29082927	0.0291	0.0044	5.71E-11	2.84E-05	Adipose Visceral Omentum
TRMT61B	ENSG00000171103.6	2	29082927	0.0444	0.0068	6.31E-11	2.93E-06	Muscle Skeletal
TRMT61B	ENSG00000171103.6	2	29082927	0.0245	0.0037	2.69E-11	3.97E-05	Adipose Subcutaneous
SPTBN1	ENSG00000115306.11	2	54790117	-0.0960	0.0118	3.15E-16	8.74E-04	Whole Blood GTEx
SPTBN1	ENSG00000115306.11	2	54790117	0.1699	0.0275	6.55E-10	2.22E-02	Adipose Subcutaneous
SPTBN1	ILMN_1690708	2	54885095	-0.2898	0.0507	1.08E-08	5.76E-05	Whole blood westra
SPTBN1	ILMN_1661335	2	54893166	-0.1694	0.0157	4.31E-27	3.28E-04	Whole blood westra
SPTBN1	ILMN_1661335	2	54893167	-0.1344	0.0130	3.13E-25	1.65E-03	Whole blood CAGE
CLHC1	ENSG00000162994.11	2	55430708	-0.0114	0.0021	1.12E-07	7.06E-03	Adipose Subcutaneous
CLHC1	ENSG00000162994.11	2	55430708	-0.0137	0.0026	1.41E-07	9.57E-04	Adipose Visceral Omentum
RFT1	ENSG00000163933.5	3	53143488	-0.0244	0.0045	7.59E-08	2.82E-02	Adipose Subcutaneous
RFT1	ENSG00000163933.5	3	53143488	-0.0222	0.0040	2.66E-08	6.61E-02	Adipose Visceral Omentum
RFT1	ENSG00000163933.5	3	53143488	-0.0296	0.0054	4.30E-08	1.28E-03	Muscle Skeletal
ZXDC	ILMN_1743643	3	126156609	0.0275	0.0050	5.14E-08	1.20E-02	Whole blood westra
ZXDC	ILMN_1743643	3	126156610	0.0278	0.0053	1.49E-07	6.16E-02	Whole blood CAGE
ZXDC	ENSG00000070476.10	3	126175603	0.0278	0.0058	1.59E-06	2.55E-01	Adipose Subcutaneous
ZXDC	ENSG00000070476.10	3	126175603	0.0245	0.0050	1.04E-06	1.41E-01	Adipose Visceral Omentum
IDUA	ENSG00000127415.8	4	989550	0.1045	0.0224	3.11E-06	7.27E-01	Muscle Skeletal
FGFRL1	ENSG00000127418.10	4	1012204	-0.2370	0.0420	1.61E-08	NA	Muscle Skeletal
RNF212	ILMN_1787259	4	1065622	0.1483	0.0296	5.57E-07	8.75E-08	Whole blood CAGE
RNF212	ENSG00000178222.8	4	1078696	0.0495	0.0104	1.78E-06	2.49E-01	Adipose Subcutaneous
RNF212	ENSG00000178222.8	4	1078696	0.0549	0.0106	2.40E-07	2.44E-01	Muscle Skeletal
UVSSA	ENSG00000163945.11	4	1361445	0.0282	0.0046	5.90E-10	9.33E-06	Liver
PJA2	ENSG00000198961.5	5	108708052	-0.0996	0.0208	1.58E-06	8.06E-01	Adipose Subcutaneous
PJA2	ENSG00000198961.5	5	108708052	-0.1740	0.0343	3.76E-07	6.27E-01	Whole Blood GTEx
BTN3A2	ILMN_1700067	6	26368278	-0.0118	0.0019	6.36E-10	2.32E-04	Whole blood CAGE
BTN3A2	ENSG00000186470.9	6	26371966	-0.0144	0.0024	3.45E-09	1.40E-03	Adipose Subcutaneous
BTN3A2	ENSG00000186470.9	6	26371966	-0.0120	0.0023	2.18E-07	5.06E-03	Ovary
BTN3A2	ENSG00000186470.9	6	26371966	-0.0130	0.0024	1.02E-07	8.13E-01	Liver
BTN3A2	ENSG00000186470.9	6	26371966	-0.0155	0.0026	2.78E-09	9.14E-03	Whole Blood GTEx
BTN3A2	ENSG00000186470.9	6	26371966	-0.0137	0.0023	2.15E-09	2.43E-02	Muscle Skeletal
BTN3A2	ENSG00000186470.9	6	26371966	-0.0164	0.0028	2.78E-09	5.01E-04	Adipose Visceral Omentum
BTN3A2	ILMN_1676528	6	26378138	-0.0123	0.0020	6.68E-10	6.96E-05	Whole blood CAGE
ZKSCAN4	ILMN_1804571	6	28212601	0.0395	0.0081	9.44E-07	5.76E-03	Whole blood CAGE

HLA-A	ENSG00000206503.7	6	29911349	-0.0420	0.0075	1.89E-08	1.80E-01	Whole Blood GTEx
HLA-A	ENSG00000206503.7	6	29911349	0.0226	0.0047	1.43E-06	1.44E-07	Ovary
HLA-A	ENSG00000206503.7	6	29911349	-0.0483	0.0078	6.89E-10	2.61E-02	Liver
EPDR1	ENSG00000086289.7	7	37975853	0.2048	0.0412	6.53E-07	5.19E-09	Muscle Skeletal
EPDR1	ENSG00000086289.7	7	37975853	0.1279	0.0238	8.06E-08	NA	Adipose Visceral Omentum
EPDR1	ENSG00000086289.7	7	37975853	0.1070	0.0167	1.66E-10	6.85E-08	Adipose Subcutaneous
PILRA	ENSG00000085514.11	7	99984393	-0.0121	0.0020	9.98E-10	9.21E-02	Muscle Skeletal
PILRA	ENSG00000085514.11	7	99984393	-0.0177	0.0030	3.91E-09	1.48E-02	Adipose Subcutaneous
PILRA	ENSG00000085514.11	7	99984393	-0.0110	0.0020	2.41E-08	1.11E-01	Liver
PILRA	ENSG00000085514.11	7	99984393	-0.0213	0.0038	1.52E-08	4.62E-02	Adipose Visceral Omentum
PILRA	ENSG00000085514.11	7	99984393	-0.0134	0.0025	9.67E-08	1.21E-01	Ovary
SLC4A2	ILMN_2078389	7	150772841	-0.1028	0.0223	3.91E-06	6.71E-01	Whole blood CAGE
ABCF2	ILMN_1781999	7	150911205	0.0578	0.0119	1.29E-06	4.40E-05	Whole blood CAGE
ABCF2	ENSG00000033050.3	7	150914619	0.0659	0.0135	1.14E-06	1.25E-01	Whole Blood GTEx
FAM86B3P	ENSG00000173295.3	8	8094252	0.0523	0.0051	9.17E-25	2.38E-02	Adipose Visceral Omentum
FAM86B3P	ENSG00000173295.3	8	8094252	0.0996	0.0165	1.61E-09	2.21E-01	Adipose Subcutaneous
ERI1	ENSG00000104626.10	8	8916956	-0.0740	0.0119	5.21E-10	3.05E-05	Whole Blood GTEx
PPP1R3B	ILMN_1712236	8	8996073	0.1563	0.0206	2.92E-14	1.77E-04	Whole blood westra
PPP1R3B	ILMN_1712236	8	8996074	0.1616	0.0301	8.24E-08	3.19E-02	Whole blood CAGE
MSRA	ILMN_2228180	8	10286184	-0.0909	0.0066	8.78E-44	1.23E-04	Whole blood westra
MSRA	ILMN_2228180	8	10286185	-0.0796	0.0066	5.68E-34	1.21E-04	Whole blood CAGE
RP1L1	ENSG00000183638.5	8	10516778	0.0446	0.0087	3.13E-07	1.05E-04	Adipose Subcutaneous
XKR6	ILMN_1724762	8	11058127	-0.2024	0.0293	4.88E-12	2.38E-03	Whole blood westra
XKR6	ILMN_1724762	8	11058128	-0.1956	0.0365	8.46E-08	1.00E-01	Whole blood CAGE
MTMR9	ENSG00000104643.5	8	11163785	0.1232	0.0176	2.47E-12	4.65E-05	Muscle Skeletal
MTMR9	ILMN_1652521	8	11185454	0.2427	0.0411	3.54E-09	6.45E-03	Whole blood westra
MTMR9	ILMN_1652521	8	11185455	0.1650	0.0256	1.23E-10	1.60E-01	Whole blood CAGE
FDFT1	ENSG00000079459.8	8	11674950	0.0608	0.0121	5.25E-07	2.09E-02	Adipose Visceral Omentum
FDFT1	ENSG00000079459.8	8	11674950	0.0934	0.0160	5.25E-09	1.07E-01	Adipose Subcutaneous
FDFT1	ENSG00000079459.8	8	11674950	-0.0717	0.0143	5.54E-07	5.35E-05	Muscle Skeletal
FDFT1	ILMN_1741096	8	11696256	0.0397	0.0044	2.36E-19	4.62E-08	Whole blood westra
FDFT1	ILMN_1741096	8	11696257	0.0357	0.0034	3.63E-25	1.72E-03	Whole blood CAGE
FDFT1	ILMN_2144088	8	11696385	0.0255	0.0027	9.36E-21	1.51E-07	Whole blood westra
FDFT1	ILMN_2144088	8	11696386	0.0243	0.0026	4.05E-21	8.88E-08	Whole blood CAGE
CTSB	ILMN_2359742	8	11700489	-0.0223	0.0034	3.22E-11	0.00E+00	Whole blood westra
CTSB	ILMN_2359742	8	11700490	-0.0240	0.0034	1.81E-12	2.23E-42	Whole blood CAGE
CTSB	ILMN_1696360	8	11700938	0.0375	0.0036	2.50E-25	6.72E-41	Whole blood CAGE
CTSB	ENSG00000164733.16	8	11713495	-0.1378	0.0226	1.15E-09	4.40E-01	Adipose Subcutaneous
CTSB	ENSG00000164733.16	8	11713495	-0.1768	0.0351	4.63E-07	4.28E-01	Adipose Visceral Omentum
CDK9	ILMN_1747556	9	130552088	0.0656	0.0109	1.77E-09	1.28E-02	Whole blood westra
KLF6	ILMN_1700727	10	3818466	0.0654	0.0088	9.02E-14	1.91E-07	Whole blood westra
KLF6	ILMN_1700727	10	3818467	0.0614	0.0093	3.98E-11	6.22E-07	Whole blood CAGE
BTBD16	ENSG00000138152.7	10	124064249	-0.0225	0.0035	2.25E-10	9.20E-02	Adipose Visceral Omentum
BTBD16	ENSG00000138152.7	10	124064249	-0.0210	0.0043	1.12E-06	1.95E-01	Ovary
BTBD16	ENSG00000138152.7	10	124064249	-0.0320	0.0052	5.19E-10	3.33E-01	Muscle Skeletal
BTBD16	ENSG00000138152.7	10	124064249	-0.0186	0.0026	9.61E-13	3.81E-02	Adipose Subcutaneous
NRIP3	ILMN_1759563	11	9004438	0.0707	0.0140	3.98E-07	5.35E-10	Whole blood westra
GAL	ENSG00000069482.6	11	68454945	-0.0297	0.0051	6.35E-09	2.17E-01	Whole Blood GTEx
GAL	ILMN_1682015	11	68457385	-0.1008	0.0181	2.49E-08	1.41E-01	Whole blood westra
MPHOSPH9	ILMN_1654421	12	123645687	-0.0645	0.0111	5.74E-09	3.00E-01	Whole blood CAGE
C12orf65	ENSG00000130921.3	12	123729984	0.0680	0.0135	4.54E-07	7.01E-01	Adipose Visceral Omentum

SBNO1	ILMN_1739943	12	123782563	0.0894	0.0180	6.84E-07	6.27E-01	Whole blood CAGE
DOCK9	ENSG00000088387.13	13	99592310	0.0850	0.0145	4.84E-09	3.49E-01	Whole Blood GTEx
DOCK9	ENSG00000088387.13	13	99592310	-0.0950	0.0192	7.24E-07	9.66E-01	Muscle Skeletal
SRP54	ILMN_1753862	14	35497375	-0.0376	0.0058	7.90E-11	4.49E-13	Whole blood CAGE
SRP54	ILMN_2312275	14	35498322	-0.0353	0.0054	4.64E-11	5.32E-15	Whole blood CAGE
XRCC3	ENSG00000126215.9	14	104172893	0.0493	0.0086	9.27E-09	9.04E-05	Whole Blood GTEx
XRCC3	ENSG00000126215.9	14	104172893	0.0651	0.0121	8.33E-08	2.00E-01	Muscle Skeletal
XRCC3	ENSG00000126215.9	14	104172893	0.0556	0.0103	6.07E-08	7.50E-02	Adipose Subcutaneous
CYP19A1	ENSG00000137869.9	15	51565530	0.1436	0.0235	9.75E-10	2.41E-01	Whole Blood GTEx
PKD1	ILMN_2339028	16	2139150	-0.0789	0.0137	8.84E-09	9.92E-01	Whole blood CAGE
PKD1	ENSG00000008710.13	16	2162308	-0.0989	0.0211	2.84E-06	4.45E-02	Adipose Subcutaneous
TNRC6A	ILMN_1714622	16	24835531	0.0321	0.0059	4.88E-08	3.68E-01	Whole blood CAGE
G6PC3	ENSG00000141349.4	17	42150906	0.1259	0.0211	2.35E-09	2.13E-08	Whole Blood GTEx
G6PC3	ENSG00000141349.4	17	42150906	0.1133	0.0173	6.09E-11	1.42E-08	Muscle Skeletal
G6PC3	ILMN_2127477	17	42153390	0.2335	0.0400	5.24E-09	1.35E-01	Whole blood westra
G6PC3	ILMN_2127477	17	42153391	0.1207	0.0213	1.40E-08	5.29E-02	Whole blood CAGE
NMT1	ENSG00000136448.7	17	43157682	0.0952	0.0166	9.33E-09	1.33E-01	Adipose Visceral Omentum
NMT1	ENSG00000136448.7	17	43157682	0.0673	0.0109	6.77E-10	1.06E-03	Adipose Subcutaneous
NMT1	ILMN_1762678	17	43186237	0.0987	0.0178	3.02E-08	5.39E-03	Whole blood westra
NMT1	ILMN_1762678	17	43186238	0.0688	0.0121	1.45E-08	3.05E-03	Whole blood CAGE
PLEKHM1	ENSG00000225190.4	17	43540690	-0.0701	0.0094	1.03E-13	5.73E-01	Muscle Skeletal
PLEKHM1	ENSG00000225190.4	17	43540690	-0.0822	0.0166	7.23E-07	4.12E-02	Adipose Subcutaneous
CRHR1	ENSG00000120088.10	17	43806230	-0.0448	0.0070	1.50E-10	9.75E-01	Adipose Visceral Omentum
CRHR1	ENSG00000120088.10	17	43806230	-0.0493	0.0087	1.43E-08	1.00E+00	Adipose Subcutaneous
KANSL1	ENSG00000120071.8	17	44205007	-0.0545	0.0088	7.43E-10	1.82E-01	Muscle Skeletal
KANSL1	ENSG00000120071.8	17	44205007	-0.0436	0.0089	9.39E-07	7.15E-07	Whole Blood GTEx
DDX5	ILMN_1805344	17	62496088	0.0318	0.0039	2.53E-16	3.19E-01	Whole blood westra
DDX5	ILMN_1805344	17	62496089	0.0309	0.0039	4.66E-15	NA	Whole blood CAGE
DDX5	ENSG00000108654.7	17	62500025	0.0553	0.0092	2.07E-09	NA	Adipose Subcutaneous
DDX5	ENSG00000108654.7	17	62500025	0.0569	0.0091	4.51E-10	NA	Whole Blood GTEx
ASPSCR1	ENSG00000169696.11	17	79954982	0.0498	0.0102	1.01E-06	8.63E-01	Muscle Skeletal
ASPSCR1	ILMN_1660749	17	79975259	-0.0303	0.0058	1.77E-07	6.90E-04	Whole blood CAGE
SSBP4	ENSG00000130511.11	19	18537435	-0.0547	0.0101	6.83E-08	4.56E-03	Muscle Skeletal
NCOA6	ILMN_1695797	20	33302950	0.1026	0.0204	4.88E-07	2.10E-07	Whole blood CAGE
TRPC4AP	ILMN_2402805	20	33590403	-0.0312	0.0031	4.24E-24	5.62E-21	Whole blood westra
TRPC4AP	ILMN_2402806	20	33590649	0.0523	0.0073	5.42E-13	1.59E-08	Whole blood westra
TRPC4AP	ILMN_2402806	20	33590650	0.0454	0.0052	4.27E-18	1.48E-07	Whole blood CAGE
ZBED4	ILMN_1782129	22	50281654	-0.0732	0.0143	3.31E-07	3.26E-02	Whole blood westra
ZBED4	ILMN_1782129	22	50281655	-0.0571	0.0114	5.57E-07	3.41E-02	Whole blood CAGE
CRELD2	ILMN_1748707	22	50321003	-0.0162	0.0032	5.95E-07	1.19E-05	Whole blood westra
CRELD2	ILMN_1748707	22	50321004	-0.0168	0.0034	9.28E-07	4.57E-06	Whole blood CAGE

Supplementary Table S4 RNAm_SNPs affected circulating protein levels

SNP	Chr	position	Protein.coding.Ge	Protein	Allele1	Allele2	Effect	StdErr	P_pQTL	Study
rs807936	3	47322496	CCL4	MIP1b	T	C	-0.0849	0.0161	1.27E-07	Ahola-Olli et al
rs807931	3	47389317	CCL4	MIP1b	T	C	-0.0845	0.0161	1.42E-07	Ahola-Olli et al
rs6442112	3	48316034	MST1	Hepatocyte grc	A	G	0.3507	0.0458	4.66E-14	Suhre et al.
rs2645429	8	11660051	CTSB	Cathepsin B	T	G	0.9150	0.0200	1.00E-300	Suhre et al.
rs7513688	1	6652745	PLXNC1	PLXNC1.4564	A	G	0.1245	0.0257	1.23E-06	Sun et al.
rs2294532	1	6694927	LRTM2	LRTM2.8906.(	T	C	-0.1077	0.0259	3.31E-05	Sun et al.
rs2294532	1	6694927	MRC2	MRC2.3041.5	T	C	0.1082	0.0259	3.02E-05	Sun et al.
rs2294532	1	6694927	PLXNC1	PLXNC1.4564	T	C	0.1121	0.0259	1.55E-05	Sun et al.
rs430600	1	89225976	CCL1	CCL1.13687.5	T	C	0.1070	0.0252	2.19E-05	Sun et al.
rs430600	1	89225976	FGFR3	FGFR3.13669.	T	C	0.1042	0.0252	3.63E-05	Sun et al.
rs430600	1	89225976	CCBL2	CCBL2.12682	T	C	-0.1185	0.0252	2.57E-06	Sun et al.
rs430600	1	89225976	IGFL4	IGFL4.6353.6(	T	C	0.1075	0.0252	2.00E-05	Sun et al.
rs6673179	1	89416516	CCBL2	CCBL2.12682	T	C	0.1687	0.0246	6.46E-12	Sun et al.
rs910697	1	113063125	ST6GALNAC3	ST6GALNAC3	A	G	0.1026	0.0245	2.82E-05	Sun et al.
rs910697	1	113063125	FLT3LG	FLT3LG.1409	A	G	0.1040	0.0245	2.24E-05	Sun et al.
rs910697	1	113063125	CBFB	CBFB.10048.7	A	G	0.1006	0.0245	4.07E-05	Sun et al.
rs6682737	1	113136229	FLT3LG	FLT3LG.1409	T	C	0.1072	0.0241	8.91E-06	Sun et al.
rs6682737	1	113136229	CRYAA	CRYAA.1008	T	C	-0.0992	0.0241	3.98E-05	Sun et al.
rs6682737	1	113136229	ST6GALNAC3	ST6GALNAC3	T	C	0.1067	0.0241	9.77E-06	Sun et al.
rs6682737	1	113136229	CBFB	CBFB.10048.7	T	C	0.1001	0.0241	3.39E-05	Sun et al.
rs2974935	1	155181843	NPPB	NPPB.7655.11	T	G	-0.1120	0.0246	5.37E-06	Sun et al.
rs2974935	1	155181843	TFF1	TFF1.9185.15.	T	G	-0.1306	0.0246	1.10E-07	Sun et al.
rs1061160	1	220154768	G6B	G6B.9466.43.3	T	C	-0.1080	0.0263	3.98E-05	Sun et al.
rs807936	3	47322496	TDGF1	TDGF1.5810.2	T	C	-0.1869	0.0252	1.20E-13	Sun et al.
rs807931	3	47389317	TDGF1	TDGF1.5810.2	T	C	-0.1856	0.0252	1.82E-13	Sun et al.
rs6442112	3	48316034	TXNDC12	TXNDC12.481	A	G	-0.1962	0.0279	2.00E-12	Sun et al.
rs6442112	3	48316034	PRDM1	PRDM1.14197	A	G	0.2175	0.0278	5.62E-15	Sun et al.
rs6442112	3	48316034	TNS2	TNS2.11667.2	A	G	0.1204	0.0280	1.74E-05	Sun et al.
rs6442112	3	48316034	DOCK9	DOCK9.14002	A	G	0.1348	0.0280	1.48E-06	Sun et al.
rs6442112	3	48316034	STOM	STOM.8261.5	A	G	0.1440	0.0280	2.69E-07	Sun et al.
rs6442112	3	48316034	TMPRSS11D	TMPRSS11D.(	A	G	0.2060	0.0279	1.48E-13	Sun et al.
rs9860285	3	49355374	ADH1B	ADH1B.9834.(	T	G	-0.1540	0.0278	2.95E-08	Sun et al.
rs9860285	3	49355374	CCL19	CCL19.4922.1	T	G	-0.1165	0.0278	2.88E-05	Sun et al.
rs9860285	3	49355374	TNS2	TNS2.11667.2	T	G	-0.1790	0.0277	1.07E-10	Sun et al.
rs9860285	3	49355374	PRDM1	PRDM1.14197	T	G	-0.3005	0.0274	6.03E-28	Sun et al.
rs9860285	3	49355374	TXNDC12	TXNDC12.481	T	G	0.2736	0.0275	2.57E-23	Sun et al.
rs9860285	3	49355374	STOM	STOM.8261.5	T	G	-0.1726	0.0277	4.90E-10	Sun et al.
rs9860285	3	49355374	DOCK9	DOCK9.14002	T	G	-0.1406	0.0278	4.27E-07	Sun et al.
rs9860285	3	49355374	TMPRSS11D	TMPRSS11D.(	T	G	-0.2009	0.0277	4.07E-13	Sun et al.
rs7432266	3	57673911	COLGALT1	COLGALT1.5(	T	C	0.1368	0.0330	3.31E-05	Sun et al.
rs75072999	4	844781	IDUA	IDUA.3169.70	A	G	0.1754	0.0429	4.27E-05	Sun et al.
rs75072999	4	844781	SSB	SSB.13526.5.3	A	G	-0.1761	0.0429	3.98E-05	Sun et al.
rs6815946	4	995305	IDUA	IDUA.3169.70	T	C	-0.3791	0.0336	1.58E-29	Sun et al.
rs115790973	4	996560	IDUA	IDUA.3169.70	C	G	-0.3836	0.0339	1.02E-29	Sun et al.
rs11247975	4	1165130	IDUA	IDUA.3169.70	T	G	-0.1459	0.0267	4.47E-08	Sun et al.
rs11538062	4	1165764	DHX8	DHX8.11601.2	T	C	0.1569	0.0357	1.10E-05	Sun et al.
rs11538062	4	1165764	COX7A1	COX7A1.8390	T	C	0.1570	0.0357	1.10E-05	Sun et al.
rs11538062	4	1165764	IDUA	IDUA.3169.70	T	C	0.2072	0.0356	6.03E-09	Sun et al.
rs6556919	5	95699865	PCSK1	PCSK1.13388.	A	C	0.4622	0.0251	1.70E-75	Sun et al.

rs1045706	5	108714298	F8	F8.13499.30.3	T	C	0.1039	0.0254	4.27E-05	Sun et al.
rs6595440	5	122718736	PGRMC2	PGRMC2.868	C	G	-0.1162	0.0250	3.47E-06	Sun et al.
rs6595440	5	122718736	LINGO1	LINGO1.6620.	C	G	-0.1181	0.0250	2.34E-06	Sun et al.
rs11958836	5	122738322	PGRMC2	PGRMC2.868	A	G	-0.1181	0.0251	2.51E-06	Sun et al.
rs11958836	5	122738322	LINGO1	LINGO1.6620.	A	G	-0.1210	0.0251	1.38E-06	Sun et al.
rs2073531	6	26375256	TAPBPL	TAPBPL.6364	A	G	-0.1828	0.0359	3.47E-07	Sun et al.
rs2073531	6	26375256	RACGAP1	RACGAP1.13	A	G	-0.1731	0.0359	1.41E-06	Sun et al.
rs2073531	6	26375256	C4A	C4A.C4B.448	A	G	0.2023	0.0359	1.70E-08	Sun et al.
rs2073531	6	26375256	GRIA4	GRIA4.10760.	A	G	0.1982	0.0359	3.31E-08	Sun et al.
rs2073531	6	26375256	PRSS3	PRSS3.3479.7	A	G	-0.1659	0.0359	3.80E-06	Sun et al.
rs2073531	6	26375256	PDE4D	PDE4D.5255.	A	G	0.2286	0.0358	1.70E-10	Sun et al.
rs2073531	6	26375256	DEFB119	DEFB119.134	A	G	0.1529	0.0359	2.09E-05	Sun et al.
rs2073531	6	26375256	MICB	MICB.5102.5	A	G	0.3225	0.0356	1.32E-19	Sun et al.
rs3757138	6	26376103	C4A	C4A.C4B.448	A	G	0.2023	0.0358	1.62E-08	Sun et al.
rs3757138	6	26376103	RACGAP1	RACGAP1.13	A	G	-0.1723	0.0359	1.55E-06	Sun et al.
rs3757138	6	26376103	PDE4D	PDE4D.5255.	A	G	0.2289	0.0358	1.55E-10	Sun et al.
rs3757138	6	26376103	GRIA4	GRIA4.10760.	A	G	0.1977	0.0358	3.39E-08	Sun et al.
rs3757138	6	26376103	MICB	MICB.5102.5	A	G	0.3219	0.0356	1.38E-19	Sun et al.
rs3757138	6	26376103	PRSS3	PRSS3.3479.7	A	G	-0.1656	0.0358	3.80E-06	Sun et al.
rs3757138	6	26376103	TAPBPL	TAPBPL.6364	A	G	-0.1823	0.0358	3.63E-07	Sun et al.
rs3757138	6	26376103	DEFB119	DEFB119.134	A	G	0.1529	0.0359	2.04E-05	Sun et al.
rs56405707	6	27640246	PRSS3	PRSS3.3479.7	A	G	0.1987	0.0373	1.00E-07	Sun et al.
rs56405707	6	27640246	TAPBPL	TAPBPL.6364	A	G	0.1559	0.0374	3.09E-05	Sun et al.
rs56405707	6	27640246	PDE4D	PDE4D.5255.	A	G	-0.3010	0.0371	5.37E-16	Sun et al.
rs56405707	6	27640246	CD96	CD96.9735.44	A	G	-0.1773	0.0374	2.09E-06	Sun et al.
rs56405707	6	27640246	GRIA4	GRIA4.10760.	A	G	-0.2549	0.0373	7.76E-12	Sun et al.
rs56405707	6	27640246	HLA-DQA2	HLA.DQA2.7	A	G	-0.1628	0.0374	1.35E-05	Sun et al.
rs56405707	6	27640246	RACGAP1	RACGAP1.13	A	G	0.1821	0.0374	1.10E-06	Sun et al.
rs56405707	6	27640246	CRISP2	CRISP2.9282.	A	G	-0.1585	0.0374	2.29E-05	Sun et al.
rs56405707	6	27640246	C4A	C4A.C4B.448	A	G	-0.2447	0.0373	5.25E-11	Sun et al.
rs56405707	6	27640246	MICB	MICB.5102.5	A	G	-0.3976	0.0369	4.17E-27	Sun et al.
rs56405707	6	27640246	DEFB119	DEFB119.134	A	G	-0.1910	0.0374	3.24E-07	Sun et al.
rs200991	6	27815494	IL19	IL19.3035.80.	A	C	-0.1342	0.0322	3.02E-05	Sun et al.
rs200991	6	27815494	GRIA4	GRIA4.10760.	A	C	-0.1535	0.0321	1.74E-06	Sun et al.
rs200991	6	27815494	PDE4D	PDE4D.5255.	A	C	-0.1939	0.0321	1.48E-09	Sun et al.
rs200991	6	27815494	DEFB119	DEFB119.134	A	C	-0.1377	0.0322	1.82E-05	Sun et al.
rs200991	6	27815494	MICB	MICB.5102.5	A	C	-0.2450	0.0320	1.74E-14	Sun et al.
rs200991	6	27815494	HLA-DQA2	HLA.DQA2.7	A	C	-0.1329	0.0322	3.55E-05	Sun et al.
rs200991	6	27815494	PRSS3	PRSS3.3479.7	A	C	0.1324	0.0321	3.72E-05	Sun et al.
rs200991	6	27815494	PDIA5	PDIA5.5593.1	A	C	0.1485	0.0321	3.80E-06	Sun et al.
rs200991	6	27815494	FN1	FN1.3435.53.2	A	C	-0.1384	0.0321	1.66E-05	Sun et al.
rs200991	6	27815494	HBEGF	HBEGF.14094	A	C	0.1446	0.0321	6.76E-06	Sun et al.
rs200991	6	27815494	C4A	C4A.C4B.448	A	C	-0.1984	0.0321	6.03E-10	Sun et al.
rs200991	6	27815494	RACGAP1	RACGAP1.13	A	C	0.1541	0.0321	1.58E-06	Sun et al.
rs9986596	6	28219661	MICB	MICB.5102.5	A	G	-0.2908	0.0330	1.23E-18	Sun et al.
rs9986596	6	28219661	CRISP2	CRISP2.9282.	A	G	-0.1467	0.0333	1.05E-05	Sun et al.
rs9986596	6	28219661	GRIA4	GRIA4.10760.	A	G	-0.1847	0.0332	2.69E-08	Sun et al.
rs9986596	6	28219661	C4A	C4A.C4B.448	A	G	-0.1845	0.0332	2.75E-08	Sun et al.
rs9986596	6	28219661	PDIA5	PDIA5.5593.1	A	G	0.1435	0.0333	1.62E-05	Sun et al.
rs9986596	6	28219661	HBEGF	HBEGF.14094	A	G	0.1455	0.0333	1.23E-05	Sun et al.
rs9986596	6	28219661	PDE4D	PDE4D.5255.	A	G	-0.2289	0.0331	4.90E-12	Sun et al.

rs9986596	6	28219661	DEFB119	DEFB119.134	A	G	-0.1521	0.0333	4.79E-06	Sun et al.
rs9986596	6	28219661	RACGAP1	RACGAP1.13	A	G	0.1691	0.0332	3.63E-07	Sun et al.
rs9986596	6	28219661	PRSS3	PRSS3.3479.7	A	G	0.1734	0.0332	1.74E-07	Sun et al.
rs9986596	6	28219661	AKT2	AKT2.14685.1	A	G	0.1373	0.0333	3.72E-05	Sun et al.
rs9986596	6	28219661	C4B	C4A.C4B.448	A	G	-0.1845	0.0332	2.75E-08	Sun et al.
rs853678	6	28297313	DEFB119	DEFB119.134	A	T	-0.1507	0.0330	4.90E-06	Sun et al.
rs853678	6	28297313	MICB	MICB.5102.5	A	T	-0.2926	0.0327	3.47E-19	Sun et al.
rs853678	6	28297313	C4A	C4A.C4B.448	A	T	-0.1696	0.0329	2.63E-07	Sun et al.
rs853678	6	28297313	GRIA4	GRIA4.10760.	A	T	-0.1749	0.0329	1.07E-07	Sun et al.
rs853678	6	28297313	PRSS3	PRSS3.3479.7	A	T	0.1770	0.0329	7.24E-08	Sun et al.
rs853678	6	28297313	RACGAP1	RACGAP1.13	A	T	0.1824	0.0329	2.95E-08	Sun et al.
rs853678	6	28297313	PDE4D	PDE4D.5255.	A	T	-0.2312	0.0328	1.82E-12	Sun et al.
rs853678	6	28297313	CRISP2	CRISP2.9282.	A	T	-0.1351	0.0330	4.17E-05	Sun et al.
rs853678	6	28297313	HBEGF	HBEGF.14094	A	T	0.1402	0.0330	2.09E-05	Sun et al.
rs2523753	6	29734727	GRIA4	GRIA4.10760.	A	G	-0.1029	0.0250	3.89E-05	Sun et al.
rs2523753	6	29734727	HLA-DQA2	HLA.DQA2.7	A	G	-0.1349	0.0250	6.31E-08	Sun et al.
rs2523753	6	29734727	TAPBPL	TAPBPL.6364	A	G	0.1330	0.0250	9.77E-08	Sun et al.
rs1061815	6	29913374	DEFB119	DEFB119.134	C	G	0.1796	0.0307	4.90E-09	Sun et al.
rs1061815	6	29913374	RACGAP1	RACGAP1.13	C	G	-0.1536	0.0307	5.75E-07	Sun et al.
rs1061815	6	29913374	PRSS3	PRSS3.3479.7	C	G	-0.1587	0.0307	2.29E-07	Sun et al.
rs1061815	6	29913374	PDE4D	PDE4D.5255.	C	G	0.2416	0.0306	2.63E-15	Sun et al.
rs1061815	6	29913374	GRIA4	GRIA4.10760.	C	G	0.2351	0.0306	1.48E-14	Sun et al.
rs1061815	6	29913374	C4A	C4A.C4B.448	C	G	0.1860	0.0307	1.32E-09	Sun et al.
rs1061815	6	29913374	CD96	CD96.9735.44	C	G	0.1920	0.0307	3.80E-10	Sun et al.
rs1061815	6	29913374	HLA-DQA2	HLA.DQA2.7	C	G	0.1572	0.0307	3.16E-07	Sun et al.
rs1061815	6	29913374	MICB	MICB.5102.5	C	G	0.2274	0.0306	1.07E-13	Sun et al.
rs2517719	6	29916253	NPTX1	NPTX1.9256.	C	G	-0.1231	0.0261	2.40E-06	Sun et al.
rs2517719	6	29916253	ENTPD3	ENTPD3.4436	C	G	-0.1264	0.0261	1.32E-06	Sun et al.
rs2517719	6	29916253	GRB7	GRB7.11281.	C	G	-0.1074	0.0261	3.89E-05	Sun et al.
rs2517719	6	29916253	TAPBPL	TAPBPL.6364	C	G	-0.2067	0.0260	1.70E-15	Sun et al.
rs2517719	6	29916253	CNDP2	CNDP2.3192.	C	G	-0.1080	0.0261	3.55E-05	Sun et al.
rs2517719	6	29916253	LAYN	LAYN.2635.6	C	G	-0.1353	0.0261	2.19E-07	Sun et al.
rs2517719	6	29916253	C1QTNF9	C1QTNF9.601	C	G	-0.1192	0.0261	5.01E-06	Sun et al.
rs2517719	6	29916253	HLA-DQA2	HLA.DQA2.7	C	G	0.1259	0.0261	1.41E-06	Sun et al.
rs2517719	6	29916253	AMELX	AMELX.8578.	C	G	0.1197	0.0261	4.57E-06	Sun et al.
rs2517719	6	29916253	TNFSF8	TNFSF8.3421.	C	G	-0.1216	0.0261	3.24E-06	Sun et al.
rs2517719	6	29916253	RNMTL1	RNMTL1.958	C	G	-0.1085	0.0261	3.31E-05	Sun et al.
rs2517719	6	29916253	KCNE2	KCNE2.10427	C	G	0.1148	0.0261	1.12E-05	Sun et al.
rs2517719	6	29916253	PIK3C2A	PIK3C2A.140	C	G	-0.1116	0.0261	1.95E-05	Sun et al.
rs2517719	6	29916253	CTF1	CTF1.2889.37	C	G	-0.1081	0.0261	3.55E-05	Sun et al.
rs36019691	6	29942384	LRRC15	LRRC15.6557	T	C	-0.1126	0.0276	4.37E-05	Sun et al.
rs36019691	6	29942384	IGFL4	IGFL4.6353.6	T	C	-0.1373	0.0275	6.17E-07	Sun et al.
rs36019691	6	29942384	IGL	IGHE.IGK.IG	T	C	0.1153	0.0276	2.88E-05	Sun et al.
rs36019691	6	29942384	AMELX	AMELX.8578.	T	C	-0.1272	0.0275	3.89E-06	Sun et al.
rs36019691	6	29942384	SCGB1C1	SCGB1C1.596	T	C	-0.1330	0.0275	1.38E-06	Sun et al.
rs36019691	6	29942384	PILRA	PILRA.8683.1	T	C	-0.1192	0.0276	1.51E-05	Sun et al.
rs36019691	6	29942384	TAPBPL	TAPBPL.6364	T	C	0.1870	0.0274	9.55E-12	Sun et al.
rs36019691	6	29942384	CTBS	CTBS.6115.40	T	C	-0.1241	0.0275	6.61E-06	Sun et al.
rs36019691	6	29942384	AMBN	AMBN.6522.5	T	C	-0.1397	0.0275	3.89E-07	Sun et al.
rs36019691	6	29942384	HLA-DQA2	HLA.DQA2.7	T	C	-0.1457	0.0275	1.20E-07	Sun et al.
rs36019691	6	29942384	IGHE	IGHE.IGK.IG	T	C	0.1153	0.0276	2.88E-05	Sun et al.

rs35835721	6	29942386	TAPBPL	TAPBPL.6364	T	G	0.1884	0.0274	6.03E-12	Sun et al.
rs35835721	6	29942386	AMBN	AMBN.6522.5	T	G	-0.1415	0.0275	2.57E-07	Sun et al.
rs35835721	6	29942386	NPTX1	NPTX1.9256.7	T	G	0.1122	0.0275	4.47E-05	Sun et al.
rs35835721	6	29942386	CTBS	CTBS.6115.40	T	G	-0.1260	0.0275	4.57E-06	Sun et al.
rs35835721	6	29942386	SCGB1C1	SCGB1C1.596	T	G	-0.1350	0.0275	8.91E-07	Sun et al.
rs35835721	6	29942386	LRRC15	LRRC15.6557	T	G	-0.1118	0.0275	4.79E-05	Sun et al.
rs35835721	6	29942386	IGHE	IGHE.IGK.IGL	T	G	0.1147	0.0275	3.02E-05	Sun et al.
rs35835721	6	29942386	HLA-DQA2	HLA.DQA2.77	T	G	-0.1453	0.0275	1.20E-07	Sun et al.
rs35835721	6	29942386	PILRA	PILRA.8683.1	T	G	-0.1197	0.0275	1.35E-05	Sun et al.
rs35835721	6	29942386	IGFL4	IGFL4.6353.60	T	G	-0.1388	0.0275	4.37E-07	Sun et al.
rs35835721	6	29942386	AMELX	AMELX.8578.	T	G	-0.1268	0.0275	3.98E-06	Sun et al.
rs35835721	6	29942386	IGK	IGHE.IGK.IGL	T	G	0.1147	0.0275	3.02E-05	Sun et al.
rs2285800	6	30257497	PRSS3	PRSS3.3479.7	C	G	0.1154	0.0284	4.90E-05	Sun et al.
rs2285800	6	30257497	HLA-DQA2	HLA.DQA2.77	C	G	-0.1375	0.0284	1.29E-06	Sun et al.
rs186215159	6	35058115	LGALS7	LGALS7.9400	A	G	-0.3814	0.0887	1.70E-05	Sun et al.
rs186215159	6	35058115	TLL1	TLL1.6383.90.	A	G	-0.3892	0.0887	1.15E-05	Sun et al.
rs4715631	6	56417545	CBX7	CBX7.13027.2	T	C	0.1127	0.0270	3.02E-05	Sun et al.
rs12660627	6	74504224	CD109	CD109.3290.5	A	G	-0.4112	0.0236	8.51E-68	Sun et al.
rs7795470	7	27286383	NCAM1	NCAM1.7746.	T	C	0.4119	0.0935	1.07E-05	Sun et al.
rs28680963	7	99905475	HTN1	HTN1.10608.9	C	G	-0.3607	0.0308	1.05E-31	Sun et al.
rs7792525	7	99972122	HTN1	HTN1.10608.9	A	G	-0.3695	0.0360	9.33E-25	Sun et al.
rs6962151	7	100028484	HTN1	HTN1.10608.9	T	C	-0.3254	0.0306	1.74E-26	Sun et al.
rs2979247	8	8888948	RETN	RETN.3046.31	A	G	0.1078	0.0253	2.14E-05	Sun et al.
rs4841497	8	10985140	GPLD1	GPLD1.9500.5	A	G	0.1066	0.0252	2.29E-05	Sun et al.
rs2645429	8	11660051	CTSB	CTSB.3061.61	A	G	0.3116	0.0277	2.14E-29	Sun et al.
rs1047643	8	11660362	CTSB	CTSB.3061.61	T	C	0.1880	0.0325	7.08E-09	Sun et al.
rs8898	8	11702542	CTSB	CTSB.3061.61	T	C	0.1544	0.0258	2.34E-09	Sun et al.
rs2005617	9	33791164	KIAA1161	KIAA1161.806	T	C	0.1122	0.0252	8.32E-06	Sun et al.
rs307658	9	33941759	KIAA1161	KIAA1161.806	T	C	-0.1142	0.0252	5.89E-06	Sun et al.
rs10821035	9	95636075	ASPN	ASPN.6451.64	T	C	0.1747	0.0255	7.24E-12	Sun et al.
rs10821035	9	95636075	CD34	CD34.9023.9.3	T	C	0.1504	0.0255	3.89E-09	Sun et al.
rs41302673	9	136270538	FLT4	FLT4.2358.19.	T	G	0.3697	0.0425	3.39E-18	Sun et al.
rs41302673	9	136270538	LIFR	LIFR.5837.49.	T	G	0.1946	0.0429	5.62E-06	Sun et al.
rs41302673	9	136270538	ISLR2	ISLR2.13124.2	T	G	0.3046	0.0427	9.33E-13	Sun et al.
rs41302673	9	136270538	IL3RA	IL3RA.13744.1	T	G	0.6166	0.0416	1.20E-49	Sun et al.
rs41302673	9	136270538	TIE1	TIE1.2844.53.1	T	G	-0.1745	0.0429	4.68E-05	Sun et al.
rs41302673	9	136270538	ADAMTS13	ADAMTS13.3	T	G	-0.1768	0.0429	3.72E-05	Sun et al.
rs41302673	9	136270538	SELP	SELP.4154.57.	T	G	0.3042	0.0427	1.02E-12	Sun et al.
rs41302673	9	136270538	QSOX2	QSOX2.8397.1	T	G	-0.6454	0.0415	1.55E-54	Sun et al.
rs41302673	9	136270538	TPST2	TPST2.8024.6	T	G	-0.2465	0.0428	8.32E-09	Sun et al.
rs41302673	9	136270538	ICAM2	ICAM2.5486.7	T	G	0.2029	0.0428	2.19E-06	Sun et al.
rs41302673	9	136270538	C1GALT1C1	C1GALT1C1.5	T	G	-0.4113	0.0424	3.02E-22	Sun et al.
rs41302673	9	136270538	GOLM1	GOLM1.8983.	T	G	-0.3914	0.0424	2.82E-20	Sun et al.
rs41302673	9	136270538	ICAM5	ICAM5.5124.6	T	G	0.2040	0.0429	1.95E-06	Sun et al.
rs41302673	9	136270538	ABO	ABO.9253.52.	T	G	-0.7920	0.0407	3.16E-84	Sun et al.
rs41302673	9	136270538	KDR	KDR.3651.50.	T	G	0.3701	0.0425	3.09E-18	Sun et al.
rs41302673	9	136270538	ICAM5	ICAM5.8245.2	T	G	0.2957	0.0427	4.27E-12	Sun et al.
rs41302673	9	136270538	SELE	SELE.3470.1.2	T	G	0.8151	0.0406	1.00E-89	Sun et al.
rs41302673	9	136270538	CHST15	CHST15.4469.	T	G	0.1795	0.0429	2.82E-05	Sun et al.
rs41302673	9	136270538	FAM177A1	FAM177A1.80	T	G	0.1916	0.0429	7.94E-06	Sun et al.
rs41302673	9	136270538	TLR4	TLR4.LY96.36	T	G	0.2105	0.0428	8.91E-07	Sun et al.

rs41302673	9	136270538	MET	MET.2837.3.2	T	G	0.2372	0.0428	2.95E-08	Sun et al.
rs41302673	9	136270538	MBL2	MBL2.3000.6(	T	G	-0.2160	0.0428	4.57E-07	Sun et al.
rs41302673	9	136270538	ADGRF5	ADGRF5.640(	T	G	0.4751	0.0422	1.95E-29	Sun et al.
rs41302673	9	136270538	CDH5	CDH5.2819.2:	T	G	0.1812	0.0429	2.34E-05	Sun et al.
rs41302673	9	136270538	ICAM1	ICAM1.4342.1	T	G	0.1919	0.0429	7.59E-06	Sun et al.
rs41302673	9	136270538	GLCE	GLCE.7808.5.	T	G	-0.3592	0.0425	3.09E-17	Sun et al.
rs41302673	9	136270538	DPEP2	DPEP2.8327.2	T	G	0.1824	0.0429	2.09E-05	Sun et al.
rs41302673	9	136270538	F8	F8.13499.30.3	T	G	-0.3789	0.0425	4.57E-19	Sun et al.
rs41302673	9	136270538	INSR	INSR.3448.13.	T	G	0.3789	0.0425	4.68E-19	Sun et al.
rs41302673	9	136270538	B3GNT2	B3GNT2.7980	T	G	-0.2202	0.0428	2.75E-07	Sun et al.
rs41302673	9	136270538	KIN	KIN.14643.27.	T	G	0.2408	0.0428	1.82E-08	Sun et al.
rs41302673	9	136270538	FAM3D	FAM3D.1310:	T	G	-0.4777	0.0422	9.77E-30	Sun et al.
rs41302673	9	136270538	CD209	CD209.3029.5	T	G	-0.5027	0.0421	7.24E-33	Sun et al.
rs41302673	9	136270538	SEMA6A	SEMA6A.794:	T	G	0.1888	0.0429	1.07E-05	Sun et al.
rs739468	9	136326248	B4GALT2	B4GALT2.959	T	G	0.1542	0.0360	1.82E-05	Sun et al.
rs739468	9	136326248	B3GNT2	B3GNT2.7980	T	G	0.2432	0.0358	1.12E-11	Sun et al.
rs739468	9	136326248	ICAM5	ICAM5.5124.6	T	G	-0.2064	0.0359	8.71E-09	Sun et al.
rs739468	9	136326248	KDR	KDR.3651.50.	T	G	-0.3996	0.0354	1.32E-29	Sun et al.
rs739468	9	136326248	FAM20B	FAM20B.719\	T	G	0.1903	0.0359	1.15E-07	Sun et al.
rs739468	9	136326248	ABO	ABO.9253.52.	T	G	0.7955	0.0333	3.80E-126	Sun et al.
rs739468	9	136326248	IL6ST	IL6ST.2620.4.	T	G	-0.1518	0.0360	2.40E-05	Sun et al.
rs739468	9	136326248	TLR4	TLR4.LY96.3(	T	G	-0.1989	0.0359	3.02E-08	Sun et al.
rs739468	9	136326248	CCL28	CCL28.2890.5	T	G	-0.1538	0.0360	1.91E-05	Sun et al.
rs739468	9	136326248	B4GALT1	B4GALT1.133	T	G	0.1647	0.0359	4.57E-06	Sun et al.
rs739468	9	136326248	ADGRF5	ADGRF5.640(	T	G	-0.4829	0.0350	3.24E-43	Sun et al.
rs739468	9	136326248	CDH12	CDH12.10701	T	G	-0.1532	0.0360	2.04E-05	Sun et al.
rs739468	9	136326248	TIE1	TIE1.2844.53.	T	G	0.1811	0.0359	4.68E-07	Sun et al.
rs739468	9	136326248	LIFR	LIFR.5837.49.	T	G	-0.1901	0.0359	1.20E-07	Sun et al.
rs739468	9	136326248	ICAM2	ICAM2.5486.7	T	G	-0.2171	0.0359	1.41E-09	Sun et al.
rs739468	9	136326248	QSOX2	QSOX2.8397.	T	G	0.6515	0.0342	9.55E-81	Sun et al.
rs739468	9	136326248	TPST2	TPST2.8024.6	T	G	0.2252	0.0358	3.39E-10	Sun et al.
rs739468	9	136326248	FLT4	FLT4.2358.19.	T	G	-0.3282	0.0356	2.88E-20	Sun et al.
rs739468	9	136326248	SELP	SELP.4154.57.	T	G	-0.3119	0.0357	2.19E-18	Sun et al.
rs739468	9	136326248	SELE	SELE.3470.1.2	T	G	-0.8174	0.0331	2.04E-134	Sun et al.
rs739468	9	136326248	C1GALT1C1	C1GALT1C1.5	T	G	0.4425	0.0352	3.55E-36	Sun et al.
rs739468	9	136326248	CDH15	CDH15.5410.5	T	G	-0.1539	0.0359	1.86E-05	Sun et al.
rs739468	9	136326248	INSR	INSR.3448.13.	T	G	-0.3417	0.0356	7.24E-22	Sun et al.
rs739468	9	136326248	F8	F8.13499.30.3	T	G	0.3750	0.0355	3.98E-26	Sun et al.
rs739468	9	136326248	MBL2	MBL2.3000.6(	T	G	0.2320	0.0358	9.55E-11	Sun et al.
rs739468	9	136326248	ISLR2	ISLR2.13124.2	T	G	-0.2935	0.0357	2.00E-16	Sun et al.
rs739468	9	136326248	DPEP2	DPEP2.8327.2	T	G	-0.1487	0.0360	3.55E-05	Sun et al.
rs739468	9	136326248	GOLM1	GOLM1.8983.	T	G	0.3780	0.0354	1.48E-26	Sun et al.
rs739468	9	136326248	ICAM1	ICAM1.4342.1	T	G	-0.1722	0.0359	1.66E-06	Sun et al.
rs739468	9	136326248	SEMA6A	SEMA6A.794:	T	G	-0.1675	0.0359	3.16E-06	Sun et al.
rs739468	9	136326248	FAM3D	FAM3D.1310:	T	G	0.5037	0.0350	5.25E-47	Sun et al.
rs739468	9	136326248	THSD1	THSD1.5621.6	T	G	-0.2338	0.0358	6.76E-11	Sun et al.
rs739468	9	136326248	KIN	KIN.14643.27.	T	G	-0.2135	0.0359	2.63E-09	Sun et al.
rs739468	9	136326248	ADAMTS13	ADAMTS13.3	T	G	0.1900	0.0359	1.20E-07	Sun et al.
rs739468	9	136326248	ICAM5	ICAM5.8245.2	T	G	-0.2808	0.0357	3.80E-15	Sun et al.
rs739468	9	136326248	IL3RA	IL3RA.13744.	T	G	-0.6081	0.0345	1.10E-69	Sun et al.
rs739468	9	136326248	MET	MET.2837.3.2	T	G	-0.2186	0.0359	1.07E-09	Sun et al.

rs739468	9	136326248	CHST15	CHST15.4469.	T	G	-0.1720	0.0359	1.70E-06	Sun et al.
rs739468	9	136326248	GLCE	GLCE.7808.5.	T	G	0.3822	0.0354	3.98E-27	Sun et al.
rs739468	9	136326248	CD209	CD209.3029.5	T	G	0.5296	0.0349	4.07E-52	Sun et al.
rs739468	9	136326248	FAM3B	FAM3B.9177.	T	G	0.1855	0.0359	2.40E-07	Sun et al.
rs739468	9	136326248	LY96	TLR4.LY96.3(	T	G	-0.1989	0.0359	3.02E-08	Sun et al.
rs1838062	10	54485449	MBL2	MBL2.3000.6(	A	G	-0.3540	0.0281	2.40E-36	Sun et al.
rs10887869	10	82194689	FAM213A	FAM213A.134	A	G	0.1504	0.0247	1.20E-09	Sun et al.
rs12260218	10	94942669	SYTL4	SYTL4.11563.	T	C	-0.1031	0.0247	3.02E-05	Sun et al.
rs2421013	10	124089036	MDK	MDK.2911.27.	A	G	0.1006	0.0246	4.27E-05	Sun et al.
rs6585816	10	124101578	TAGLN	TAGLN.9756.	T	G	0.1241	0.0301	3.72E-05	Sun et al.
rs273605	11	30965855	RFFL	RFFL.14186.1.	A	T	-0.1158	0.0267	1.41E-05	Sun et al.
rs273605	11	30965855	NISCH	NISCH.12738.	A	T	-0.1121	0.0267	2.69E-05	Sun et al.
rs174537	11	61552680	MET	MET.2837.3.2	T	G	0.1203	0.0258	3.16E-06	Sun et al.
rs174537	11	61552680	IRF1	IRF1.10351.51	T	G	-0.1106	0.0258	1.82E-05	Sun et al.
rs174537	11	61552680	FAM20B	FAM20B.7198	T	G	0.1057	0.0258	4.27E-05	Sun et al.
rs174537	11	61552680	HAVCR2	HAVCR2.715.	T	G	0.1314	0.0258	3.47E-07	Sun et al.
rs174537	11	61552680	SLC9B2	SLC9B2.9088.	T	G	-0.1074	0.0258	3.24E-05	Sun et al.
rs174537	11	61552680	TBCA	TBCA.12501.1	T	G	-0.1147	0.0258	8.71E-06	Sun et al.
rs4246215	11	61564299	TBCA	TBCA.12501.1	T	G	-0.1166	0.0256	5.25E-06	Sun et al.
rs4246215	11	61564299	HAVCR2	HAVCR2.715.	T	G	0.1272	0.0256	6.76E-07	Sun et al.
rs4246215	11	61564299	IRF1	IRF1.10351.51	T	G	-0.1053	0.0256	3.98E-05	Sun et al.
rs4246215	11	61564299	S100A13	S100A13.7223	T	G	-0.1064	0.0256	3.31E-05	Sun et al.
rs4246215	11	61564299	SLC9B2	SLC9B2.9088.	T	G	-0.1041	0.0256	4.90E-05	Sun et al.
rs4246215	11	61564299	MET	MET.2837.3.2	T	G	0.1169	0.0256	5.01E-06	Sun et al.
rs1841964	12	28343033	PTHLH	PTHLH.2962.5	A	G	0.1777	0.0281	2.45E-10	Sun et al.
rs4760610	12	48418225	COL1A1	COL1A1.1114	T	C	0.1300	0.0285	5.25E-06	Sun et al.
rs7975791	12	49413486	PSMD7	PSMD7.3898.5	T	C	-0.2638	0.0617	1.91E-05	Sun et al.
rs11057192	12	123693420	MAGEA3	MAGEA3.125	T	C	0.1236	0.0302	4.17E-05	Sun et al.
rs9557132	13	99724279	NFASC	NFASC.7179.(	C	G	-0.1373	0.0305	6.92E-06	Sun et al.
rs9557132	13	99724279	MFAP4	MFAP4.5636.1	C	G	-0.1386	0.0305	5.62E-06	Sun et al.
rs9557132	13	99724279	CACNA2D3	CACNA2D3.8	C	G	-0.1326	0.0305	1.38E-05	Sun et al.
rs3181248	14	24808168	KIR2DL5A	KIR2DL5A.77	A	G	0.1041	0.0251	3.31E-05	Sun et al.
rs2273154	14	35433187	FAM177A1	FAM177A1.8(	T	C	-0.1800	0.0308	5.25E-09	Sun et al.
rs28384418	14	35452126	FAM177A1	FAM177A1.8(	A	C	-0.1917	0.0274	2.40E-12	Sun et al.
rs61217816	14	95651047	FAM107B	FAM107B.477	T	C	-0.1811	0.0433	2.88E-05	Sun et al.
rs61217816	14	95651047	COL1A1	COL1A1.1114	T	C	-0.1965	0.0433	5.62E-06	Sun et al.
rs3814816	14	95651765	COL1A1	COL1A1.1114	A	G	0.2023	0.0432	2.82E-06	Sun et al.
rs4900592	14	104177098	NETO1	NETO1.5639.4	T	G	0.1095	0.0267	4.17E-05	Sun et al.
rs4900592	14	104177098	NPTX2	NPTX2.6521.3	T	G	0.1086	0.0267	4.90E-05	Sun et al.
rs4900592	14	104177098	PGM1	PGM1.9173.21	T	G	-0.1122	0.0267	2.69E-05	Sun et al.
rs2295146	14	104199356	NPTX2	NPTX2.6521.3	T	C	0.1072	0.0255	2.57E-05	Sun et al.
rs12050772	15	51544871	CTSH	CTSH.8465.52	T	G	0.1055	0.0253	3.09E-05	Sun et al.
rs8031179	15	65952034	IGDCC4	IGDCC4.9793.	T	C	0.1034	0.0247	2.88E-05	Sun et al.
rs112865841	16	434326	HBZ	HBZ.6919.3.3	T	G	-0.2660	0.0309	7.41E-18	Sun et al.
rs1420580	16	51164608	TIMP4	TIMP4.6462.1	T	C	0.1180	0.0261	6.46E-06	Sun et al.
rs61733768	16	67213950	TPPP2	TPPP2.12800.5	A	G	-0.4251	0.0749	1.38E-08	Sun et al.
rs8053031	16	67298163	TPPP2	TPPP2.12800.5	T	G	0.4368	0.0720	1.35E-09	Sun et al.
rs143837268	16	67654677	TPPP2	TPPP2.12800.5	T	C	-0.4907	0.0947	2.24E-07	Sun et al.
rs118016432	16	67806696	TPPP2	TPPP2.12800.5	T	C	0.3161	0.0714	9.33E-06	Sun et al.
rs4450459	17	7396617	SAT2	SAT2.12524.1	A	G	-0.2802	0.0688	4.68E-05	Sun et al.
rs4791806	17	7729184	CCL3	CCL3.3040.59	A	G	-0.1406	0.0324	1.45E-05	Sun et al.

rs4791806	17	7729184	IBSP	IBSP.3415.61.	A	G	0.1342	0.0324	3.47E-05	Sun et al.
rs55809496	17	7759477	MSRA	MSRA.7137.8.	A	C	0.1130	0.0251	6.76E-06	Sun et al.
rs62623385	17	7847837	SNX1	SNX1.8807.13	A	T	-0.2927	0.0710	3.80E-05	Sun et al.
rs62066870	17	20063758	SPACA3	SPACA3.8076	A	C	0.1245	0.0254	9.77E-07	Sun et al.
rs4792891	17	43973498	DYRK3	DYRK3.4359.	T	G	-0.1060	0.0259	4.27E-05	Sun et al.
rs4792891	17	43973498	COA3	COA3.7888.5	T	G	-0.1080	0.0259	3.09E-05	Sun et al.
rs4792891	17	43973498	CDH15	CDH15.5410.	T	G	-0.1119	0.0259	1.55E-05	Sun et al.
rs4792891	17	43973498	ADCYAP1	ADCYAP1.45	T	G	-0.1147	0.0259	9.33E-06	Sun et al.
rs4792891	17	43973498	CDC42BPB	CDC42BPB.3	T	G	-0.1190	0.0259	4.27E-06	Sun et al.
rs4792891	17	43973498	ASIC4	ASIC4.6951.2	T	G	-0.1094	0.0259	2.40E-05	Sun et al.
rs1881193	17	44248769	OPALIN	OPALIN.7736.	T	C	-0.1175	0.0288	4.57E-05	Sun et al.
rs34587622	17	75398498	VRK1	VRK1.12553.	T	C	-0.1895	0.0400	2.19E-06	Sun et al.
rs34587622	17	75398498	KIF23	KIF23.5228.2	T	C	-0.1815	0.0400	5.75E-06	Sun et al.
rs3180287	19	804396	IL1R1	IL1R1.2991.9.	T	C	-0.1457	0.0359	4.90E-05	Sun et al.
rs3180287	19	804396	EPHB6	EPHB6.9261.1	T	C	-0.1464	0.0359	4.47E-05	Sun et al.
rs3180287	19	804396	PTPRD	PTPRD.9296.1	T	C	-0.1502	0.0359	2.82E-05	Sun et al.
rs3180287	19	804396	UNC5D	UNC5D.5140.	T	C	-0.1746	0.0358	1.10E-06	Sun et al.
rs3180287	19	804396	CNTNAP2	CNTNAP2.69	T	C	-0.1766	0.0358	8.32E-07	Sun et al.
rs3180287	19	804396	EFNA5	EFNA5.2615.	T	C	-0.1545	0.0359	1.66E-05	Sun et al.
rs3180287	19	804396	LRIG3	LRIG3.3322.5	T	C	-0.1605	0.0359	7.59E-06	Sun et al.
rs291700	20	31981849	LRIT3	LRIT3.11534.	T	C	-0.1123	0.0266	2.40E-05	Sun et al.
rs291700	20	31981849	ASIP	ASIP.5676.54.	T	C	0.1980	0.0264	6.61E-14	Sun et al.
rs2295354	20	33356541	ASIP	ASIP.5676.54.	T	C	0.1274	0.0250	3.47E-07	Sun et al.
rs8501	20	33590584	ASIP	ASIP.5676.54.	T	C	0.1306	0.0320	4.37E-05	Sun et al.
rs6007010	22	45782903	TAPBP	TAPBP.12378.	T	C	0.1085	0.0260	2.95E-05	Sun et al.
rs28379706	22	50728062	PLXNB2	PLXNB2.9216	T	C	-0.5312	0.0233	4.47E-115	Sun et al.
rs28455041	22	50731903	PLXNB2	PLXNB2.9216	T	C	-0.4863	0.0249	1.15E-84	Sun et al.
rs2230471	1	172411496	CD14	CD14	G	A	0.1320	0.0250	1.76E-07	Yao et al.
rs916365	1	172453953	CD14	CD14	G	T	0.1250	0.0250	6.68E-07	Yao et al.
rs41302673	9	136270538	MCAM	MCAM	T	G	0.2290	0.0440	1.76E-07	Yao et al.
rs41302673	9	136270538	IL6ST	sGP130	T	G	0.3060	0.0440	2.59E-12	Yao et al.
rs41302673	9	136270538	SELP	GMP140	T	G	0.5910	0.0440	2.61E-41	Yao et al.
rs41302673	9	136270538	ICAM1	sICAM1	T	G	0.4690	0.0440	6.62E-27	Yao et al.
rs739468	9	136326248	ICAM1	sICAM1	G	T	0.2320	0.0260	1.58E-19	Yao et al.
rs739468	9	136326248	IL6ST	sGP130	G	T	0.1440	0.0260	2.12E-08	Yao et al.
rs739468	9	136326248	SELP	GMP140	G	T	0.3190	0.0260	3.82E-35	Yao et al.
